# Pyro‐Phyllobilins: Elusive Chlorophyll Catabolites Lacking a Critical Carboxylate Function of the Natural Chlorophylls

**DOI:** 10.1002/chem.201705331

**Published:** 2018-01-31

**Authors:** Chengjie Li, Klaus Wurst, Joachim Berghold, Maren Podewitz, Klaus R. Liedl, Bernhard Kräutler

**Affiliations:** ^1^ Institute of Organic Chemistry and Centre of Molecular Biosciences University of Innsbruck Innrain 80/82 6020 Innsbruck Austria; ^2^ Institute of General, Inorganic and Theoretical Chemistry University of Innsbruck Innrain 80/82 6020 Innsbruck Austria; ^3^ Present address: Key Laboratory for Advanced Materials and Institute of, Fine Chemicals School of Chemistry and Molecular Engineering East China University of Science and Technology Meilong Rd. 130 200237 Shanghai P.R. China

**Keywords:** cycloaddition, photoisomerization, phyllobilin, porphyrinoids, self-assembly

## Abstract

A β‐keto ester grouping is a characteristic of ring E of the chlorophylls (Chls). Its presence has also reinforced the original identification of nonfluorescent Chl catabolites (NCCs) as colorless, amphiphilic phyllobilins (PBs). Polar NCCs were also detected in higher plants, in which a free carboxyl group replaced the ring E ester group. Such NCCs are surprisingly resistant to loss of this carboxyl unit, and NCCs lacking the latter, that is, pyro‐NCCs (pyNCCs), have not been reported. Intrigued by the question of the natural occurrence of pyro‐phyllobilins (pyPBs), we have prepared a representative pyNCC by decarboxylation of a natural NCC. We also converted the pyNCC into its yellow oxidation product, a pyro‐YCC (pyYCC). The solution structures of pyNCC and of pyYCC, and a crystal structure of the pyYCC methyl ester (pyYCC‐Me) were obtained. pyYCC‐Me features the same remarkable H‐bonded and π‐stacked dimer structure as the corresponding natural yellow Chl catabolite (YCC) with the ring E methyl ester group. Indeed, the latter substituent has little effect on the structure, as well as on the unique self‐assembly and photoswitch behavior of yellow PBs.

## Introduction

In higher plants chlorophyll (Chl) breakdown^1^, ^2^ produces bilin‐type linear tetrapyrroles, named phyllobilins (PBs).^3^, ^4^, ^5^ The PBs have a similar structure to the ubiquitous heme‐derived bilins.^6^, ^7^ However, PBs carry a characteristically substituted “extra” cyclopentanone moiety, derived from ring E of Chl.^3^, ^5^ Hence, PBs are congested at their “southern” *meso*‐position, a source, probably, of extra reactivity and of unusual conformational control,^8^ as is found in highly substituted porphyrin(oid)s.^9^, ^10^ The Chl‐derived methyl ester (or carboxylic acid) group at ring E of natural PBs affects their properties and may be relevant to their possible biological functions. Indeed, in higher plants a phyllobilin lacking the carboxylate function at ring E, that is, a pyro‐phyllobilin (pyPB), remains to be identified.^6^

**Scheme 1:**
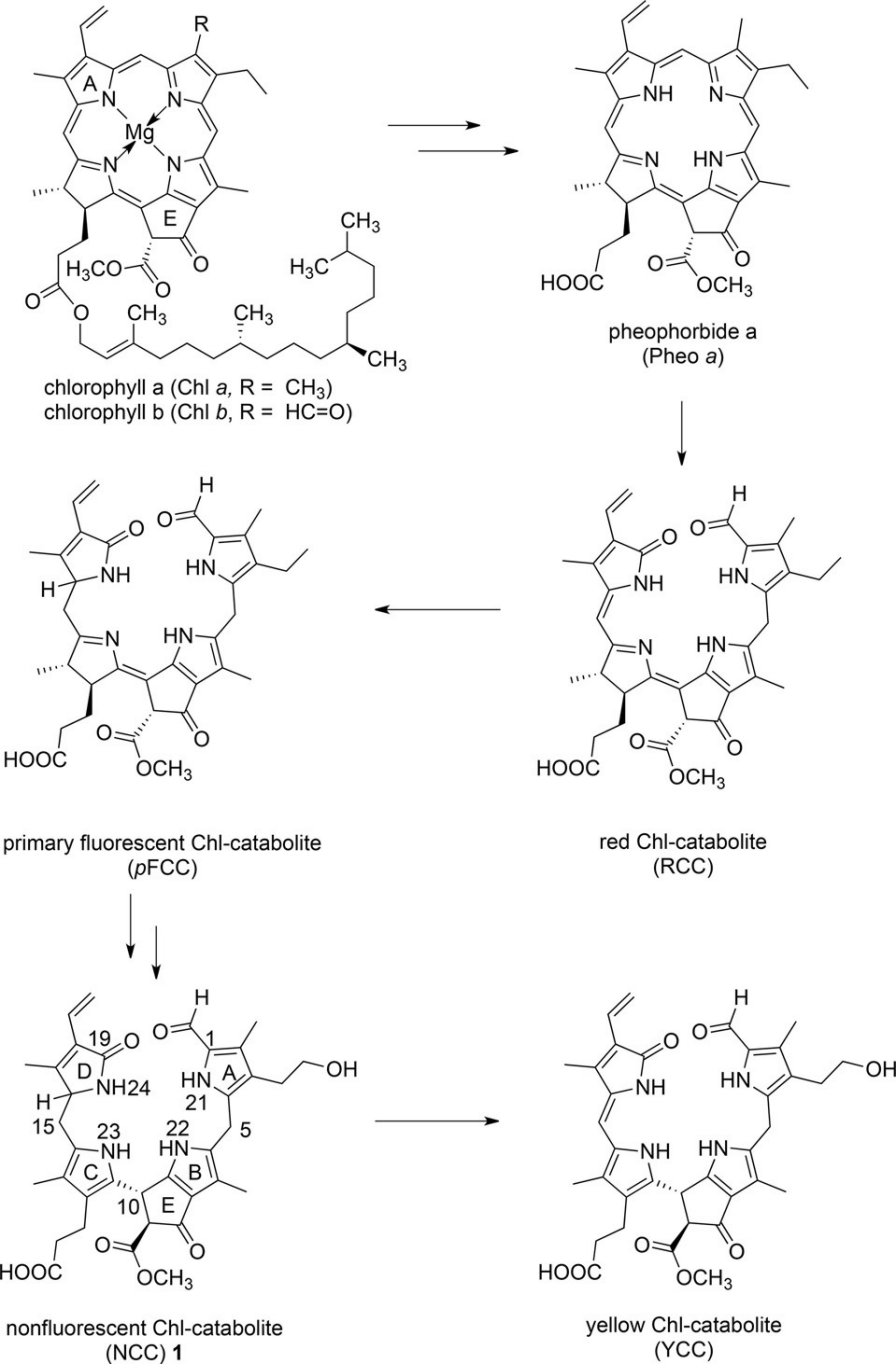
Abbreviated structural outline of the 1‐formyl‐19‐oxobilin branch of the PaO/phyllobilin pathway of Chl breakdown, which generates phyllobilins that feature the characteristic ring E methyl ester function of the Chs (as represented here), or a corresponding free carboxylic acid group.^4^, ^6^

The natural formation of PBs from Chls implies an oxidative opening at the “northern” (α) *meso*‐position of the macrocycle.^11^, ^12^ This key step is achieved by pheophorbide a oxygenase (PaO), which specifically degrades pheophorbide *a* (Pheo *a*) to the red Chl catabolite (RCC).^11^, ^12^, ^13^ This red 1‐formyl‐19‐oxo‐bilin is the precursor of further colorless products of Chl breakdown in higher plants along the common PaO/phyllobilin (PaO/PB) pathway.^4^, ^14^ Based on about 80 PB structures delineated over the years,^6^ several other key enzymes of the PaO/PB‐path have been identified in recent years.^14^ A large part of the relatively abundant and colorless “nonfluorescent” Chl catabolites (NCCs), ^[15]^ such as the NCC **1**, and *p*FCC, a “fluorescent” Chl catabolite (FCC), carry the original, Chl derived β‐keto ester substituent at their ring E moiety^6^ (see Scheme 1). Alternatively, colorless PBs may be functionalized there by a β‐keto‐carboxylic acid function.^6^, ^16^, ^17^ A cytosolic methyl esterase, first identified in *Arabidopsis thaliana* leaves, hydrolyzes the Chl‐derived methyl ester function.^18^

**Scheme 2:**
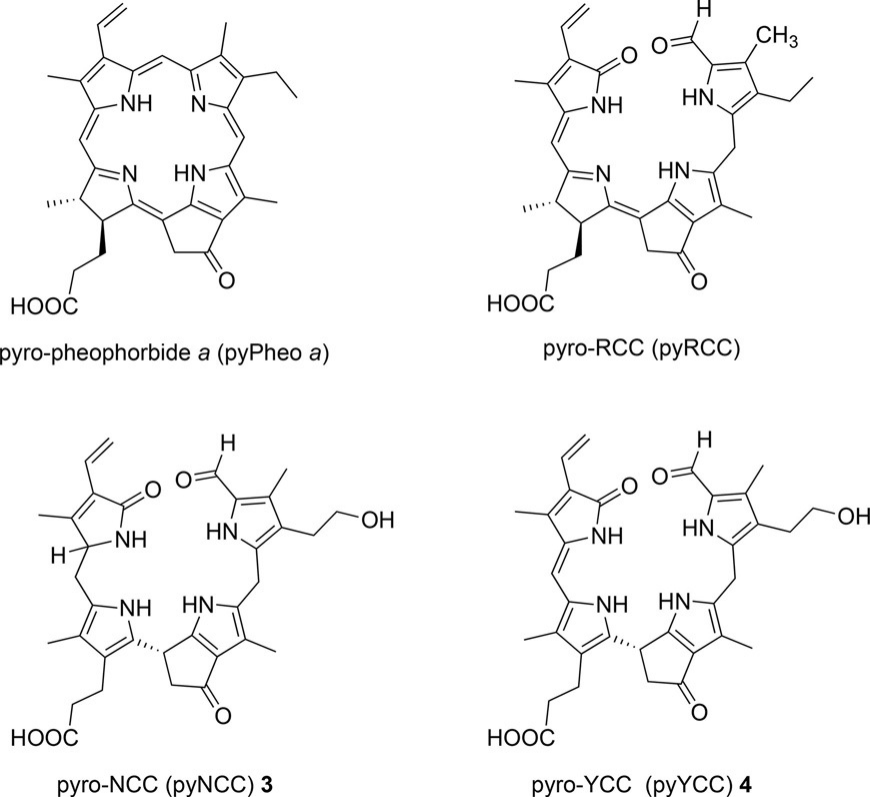
Chemical formulas of pyro‐pheophorbide *a*, of the known red pyro‐RCC^23^ and of the here introduced two types of pyro‐phyllobilins (pyPBs), the pyro‐type 1‐formyl‐19‐oxobilins pyro‐NCC **3** and pyro‐YCC **4**.

In earlier work, in extracts of the green alga *Chlamydomonas reinhardtii* 19, 20 pyro‐pheophorbide *a* (pyPheo *a*) was detected under anaerobic conditions, and a “decarboxymethylase” was suggested to be relevant in Chl breakdown in this alga (see Scheme 2). Alternatively, this product of an apparent loss of the entire methyl ester group of Pheo *a* may result from a two‐step sequence via spontaneous decarboxylation of the first‐formed 13^2^‐carboxyl‐pyro‐pheophorbide *a*.^21^, ^22^ Likewise, a red pyro‐RCC (pyRCC) was described as an adventitious decarboxylation product of its endogenous β‐keto‐carboxylic acid precursor, secreted by the bleached green alga *Auxenachlorella protothecoides*.^23^, ^24^ By chemical means this pyRCC was converted into incompletely characterized colorless linear tetrapyrroles devoid of the carboxylic acid group.^24^

We have become interested in the question of the existence of pyPBs in senescent plants, and in the consequence of the lack of the carboxylate function on the structure and chemical reactivity of pyPBs. The polar colorless PBs with a β‐keto‐carboxylic acid group, such as the NCC **2**, turned out to be remarkably resistant to decarboxylation. We report here 1) the partial synthesis of the pyro‐type NCC (pyNCC) **3** from NCC **1**, 2) oxidation of **3** to the yellow pyro‐type YCC (pyYCC) **4**, and 3) structural, spectroscopic, and photochemical properties of these novel pyPBs.

## Results and Discussion

**Scheme 3:**
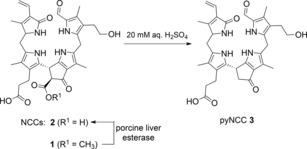
Outline of the partial synthesis of pyNCC **3** from the natural NCC **2**.

The semisynthetic pyNCC **3** was selected as a first model pyNCC (see Scheme 3). The natural polar NCC **2** represents a rational substrate for the preparation of **3** by decarboxylation. NCC **2** (named *So*‐NCC‐3 originally as it was first isolated from senescent leaves of spinach) features two free carboxylic acid functions.^6^, ^25^ In spite of the identification of the polar NCC **2** in a variety of plants,^25^, ^26^ so far, an efficient natural supply for it has not been found. Hence, the abundant NCC **1** (a 1‐formyl‐3^2^‐hydroxy‐19‐oxo‐16,19‐dihydro‐16‐*epi*‐phyllobilane) served as precursor of **2** and as the effective starting material for the semi‐synthesis of the pyNCC **3** (Scheme 3). NCC **1** was first isolated from de‐greened leaves of the deciduous Katsura trees (*Cercidiphyllum japonicum*)^27^ and named *Cj*‐NCC‐1.^28^ For the preparation of the NCC **2** with a carboxylic acid group at the 8^2^‐position (96 % yield), the NCC **1** was treated with porcine esterase (163 U mg^−1^) at 38 °C in aqueous phosphate buffer (pH 7.9) in the dark. Spectral analysis confirmed the formation of **2**, which was identified separately with *So*‐NCC‐3.^25^

At room temperature the polar NCC **2** was rather stable, even at pH 4.0. However, with 20 mm aqueous H_2_SO_4_ at 80 °C, the decarboxylation reaction of **2** proceeded within hours. To prepare pyNCC **3**, a deoxygenated solution of the NCC **2** (680 mg, 1.08 mmol) in aqueous 20 mm H_2_SO_4_ (310 mL) was heated under Ar at 80 °C for six hours. After workup (see the Experimental Section), about 560 mg of a raw mixture containing about 50 % pyNCC **3** were isolated by precipitation. A 100 mg sample of the mixture of raw **3** was further purified using preparative HPLC, furnishing two pure fractions with UV/Vis absorption and CD characteristics typical of natural NCCs. pyNCC **3** was obtained as an off‐white powder (33 mg, 56 μmol), as were 8 mg (14 μmol) of an isomer of **3**, tentatively identified as the C16‐epimer of **3**. In addition, a minor fraction with UV/Vis absorption and HPLC properties of the pyYCC **4** was also isolated.

A (+)‐ion HR FAB‐mass spectrum of **3** displayed a pseudo‐molecular ion [*M*+H]^+^ at *m*/z: 587.2870, consistent with the expected molecular formula (C_33_H_38_N_4_O_6_) of **3**. In a 500 MHz ^1^H NMR spectrum of **3** in CD_3_OD at 26 °C the signals of all 32 nonexchangeable protons could be assigned. It displayed a set of characteristic signals of typical 1‐formyl‐19‐oxophyllobilanes. However, signals at *δ*=2.72 ppm (H_A_C8^2^) and 3.27 ppm (H_B_C8^2^) of a new methylene group correlated with C8^2^ at *δ*=51.8 ppm. H_A_ and H_B_ coupled with HC10, a double doublet (*J*_AX_=2.0 Hz, *J*_BX_=6.8 Hz) at *δ*=4.58 ppm. Furthermore, a ROESY spectrum showed correlations between the signals of H_2_C12^1^ and H_2_C8^2^, suggesting H_A_C8^2^ is in a *trans*‐position to the proton at HC10 and H_B_C8^2^ in a *cis*‐position. The NMR studies (by ^1^H,^1^H‐COSY, ^1^H,^1^H‐ROESY, ^1^H,^13^C‐HSQC, and ^1^H,^13^C‐HMBC) confirmed the chemical constitution of **3** as a 1‐formyl‐3^2^‐hydroxy‐8^2^‐demethoxycarbonyl‐19‐oxo‐16,19‐dihydro‐16*epi*‐phyllobilane.

A slightly more polar, minor NCC component, of which 8 mg (14 μmol) were also isolated, was tentatively identified as an epimer of **3**. Thus, the positive ion FAB‐mass spectrum of this fraction showed a pseudo‐molecular ion [*M*+H] at *m*/*z*: 587.2, again consistent with the molecular formula C_33_H_38_N_4_O_6_. Its 500 MHz ^1^H NMR spectrum displayed considerable similarities to that of **3**. The most noticeable differences occurred for signals assigned to HC16 and H_3_C17, suggesting that this minor pyNCC component represents the C16‐epimer of **3**, that is, to be a 1‐formyl‐3^2^‐hydroxy‐8^2^‐demethoxycarbonyl‐19‐oxo‐16,19‐dihydro‐16*n*‐phyllobilane (see Refs. 3, 6 for nomenclature of PBs). A further, less polar yellow fraction was identified as pyYCC **4** on the basis of its UV/Vis‐spectrum with an absorption maximum at 432 nm, and its (+)‐ion FAB‐mass spectrum, showing pseudo‐molecular ions [*M*+K]^+^ and [*M*+H]^+^ at *m*/*z*: 623.3 and 585.3, respectively (see below for a more detailed analysis of **4**).

**Scheme 4:**
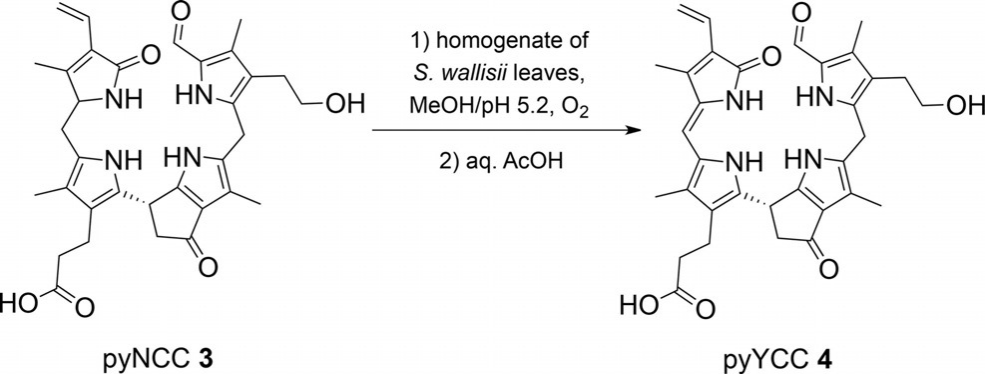
Partial synthesis of the pyYCC **4** from pyNCC **3**.

In senescent leaves yellow Chl catabolites (YCCs) have been observed.^29^ As discovered recently,^30^ homogenates of *Spathiphyllum wallisii* (*S. wallisii*) leaves oxidize NCC **1** to corresponding 15‐hydroxy‐ and/or 15‐methoxy‐substituted NCCs. These polar NCCs undergo acid‐induced elimination of H_2_O or MeOH at C15 and C16, respectively, producing YCC, identical with *Cj*‐YCC‐2, first found in extracts of senescent leaves of *Cercidiphyllum japonicum* (*C. japonicum*).^29^ Homogenates of *S. wallisii* leaves were tested here for the analogous preparative oxidation of the pyNCC **3** to the corresponding pyYCC **4** (Scheme 4). In this case, a sample of **3** (8.2 mg, 14 μmol) in 6 mL of a 1:1 mixture of MeOH and aqueous phosphate buffer pH 5.2 was treated with freshly ground leaf material from 25 cm^2^ of yellow‐green *S. wallisii* leaves. The slurry was stirred for 22 hours at 23 °C under O_2_ in the dark. Workup of the resulting reaction mixture (see the Experimental Section) furnished 4.8 mg (8.2 μmol, 59 % yield) of yellow pyYCC **4**, identified by its spectral features as described below.

**Figure 1:**
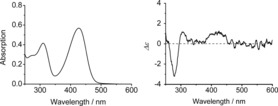
UV/Vis and CD spectra of pyYCC **4** in MeOH (1.4×10^−5^ m, 23 °C).

The UV/Vis spectrum of the pyYCC **4** in MeOH showed maxima at 427 and 310 nm (see Figure 1), very similar to those of YCC,^29^ which suggests the identity of their chromophores. Retention of configuration at the C10‐position was verified by the basically similar CD spectra of **4** and of the YCC in MeOH. The molecular formula of **4** (C_33_H_36_N_4_O_6_) was confirmed by the pseudo‐molecular ions [*M*+H]^+^, [*M*+Na]^+^, and [*M*+K]^+^ at *m*/*z*: 585.3, 607.3, and 623.3, respectively, in its (+)‐ion ESI‐mass spectrum. A MS/MS spectrum showed typical fragment ions at *m*/*z*: 567.3 [*M*‐H_2_O+H]^+^ and at *m*/*z*: 432.2 [*M*‐ring A+H]^+^.

**Figure 2:**
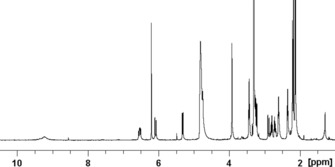
500 MHz ^1^H NMR spectrum of pyYCC **4** in CD_3_OD (25 °C).

The constitution of **4** was then deduced from NMR data. A 500 MHz ^1^H NMR spectrum of **4** (25 °C) in CD_3_OD (see Figure 2 and the Supporting Information, Table S1) showed the signals of 30 nonexchangeable hydrogen atoms. By detailed NMR analyses (^1^H,^1^H‐COSY, ^1^H,^1^H‐ROESY, ^1^H,^13^C‐HSQC, and ^1^H,^13^C‐HMBC) the complete assignment of the ^1^H and ^13^C signals of **4** was achieved. The ^1^H NMR spectrum featured the new singlet of HC15 at *δ*=6.21 ppm. A ^1^H,^1^H‐ROESY spectrum confirmed the *Z* configuration of the double bond C15=C16. A main difference between the ^1^H NMR spectra of pyYCC **4** and YCC was due to H_2_C8^2^, which gave rise to two signals at *δ*=2.89 and 3.24 ppm in the spectrum of **4**, coupling with each other and HC10 in a ^1^H,^1^H‐COSY spectrum. In the ^1^H,^13^C‐HSQC spectrum of pyYCC **4** these protons correlated with carbon atoms C8^2^ at *δ*=50.5 ppm and C10 at *δ*=32.4 ppm, whereas the corresponding carbon atoms of YCC gave signals at *δ*=67.3 and 37.1 ppm, respectively.^26^ Due to the lack of the substituent at C8^2^, the ^13^C‐signals of C10 and C8^2^ of **4** moved to higher field, when compared with those of the spectrum of YCC.

**Scheme 5:**
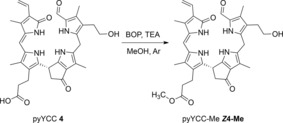
Preparation of pyYCC‐Me (***Z*** **4**‐**Me**) by esterification of pyYCC **4** (BOP=(benzotriazol‐1yl‐oxy)tris(dimethylamino)phosphonium‐hexafluorophosphate, TEA=triethylamine).

Treatment of 2.7 mg (4.6 μmol) of the pyYCC (**4**) with an excess of (benzotriazol‐1‐yl‐oxy)‐tris(dimethylamino)‐phosphonium hexafluorophosphate (BOP, 4 mg, 9.0 μmol) and triethylamine (TEA, 5 μL) in MeOH under Ar at 23 °C produced the pyYCC methyl ester (pyYCC‐Me, ***Z*** **4**‐**Me**, Scheme 5). Purification and crystallization from CHCl_3_/*n*‐C_6_H_14_ furnished 2.3 mg of yellow microcrystals of ***Z*** **4**‐**Me** (83 % yield).

The UV/Vis absorption and CD spectra of pyYCC‐Me (***Z*** **4**‐**Me**) and of pyYCC (**4**) in MeOH were very similar, indicating common chromophores and an *R* configuration at the C10 position. The derived molecular formula of ***Z*** **4**‐**Me** (C_34_H_38_N_4_O_6_) was confirmed in an ESI‐mass spectrum, with pseudo‐molecular ions [*M*+H]^+^ and [*M*+Na]^+^ at *m*/*z*: 599.3 and 621.3.

**Figure 3:**
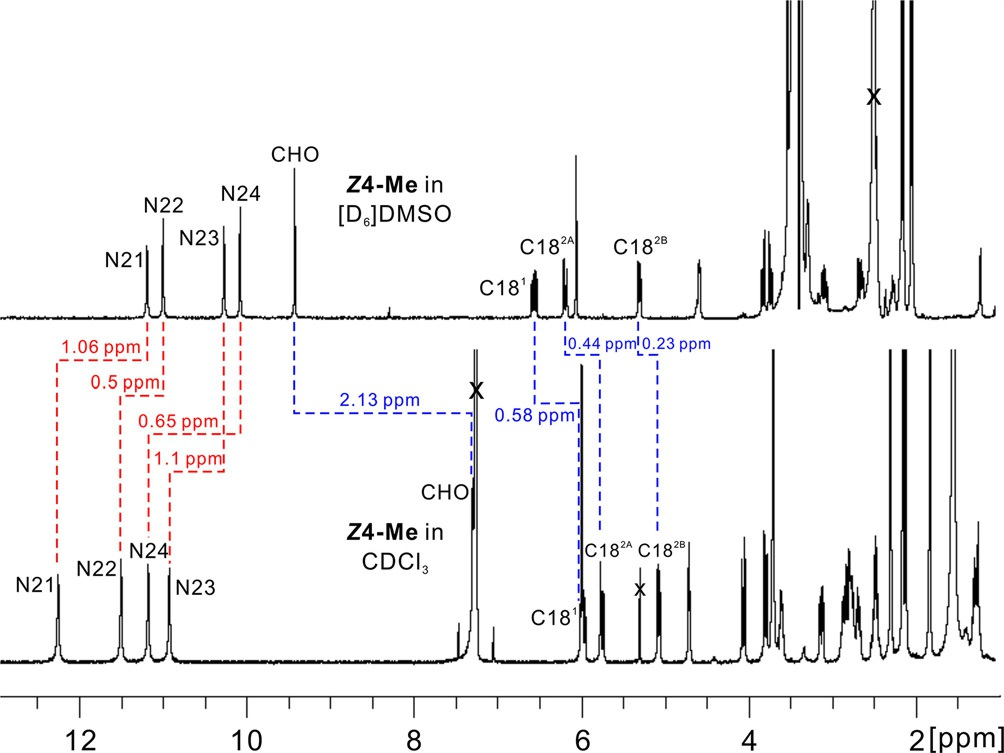
500 MHz ^1^H NMR spectra of pyYCC‐Me ***Z*** **4**‐**Me** at 25 °C and in [D_6_]DMSO (top, 6.3×10^−3^ m, 500 MHz) and in CDCl_3_ (bottom, 6.3×10^−3^ m, 500 MHz). The spectrum in [D_6_]DMSO indicates the monomeric form of ***Z*** **4**‐**Me** and that in CDCl_3_ is consistent with as extensively hydrogen‐bonded and π‐stacked dimers. The shift for the signals of NHs and CHO at low field and CH=CH_2_ at intermediate field in two solvents are labeled (red dashed line: low‐field shift with labeling of Δ*δ*; blue dashed line: high‐field shift with labeling of Δ*δ*).

The structure of the pyYCC‐Me ***Z*** **4**‐**Me** was first established by its NMR spectra. A 500 MHz ^1^H‐NMR spectrum of pyYCC‐Me at 25 °C in [D_6_]DMSO (Figure 3) revealed the signals of all its 38 hydrogen atoms. At low field five signals of a formyl and of four pyrrole NH atoms were present, the signature of a peripheral CH=CH_2_ group and the singlet of HC15. From thorough NMR analyses (^1^H,^1^H‐COSY, ^1^H,^1^H‐ROESY, ^1^H,^13^C‐HSQC, and ^1^H,^13^C‐HMBC) the complete assignment of the ^1^H and ^13^C signals of ***Z*** **4**‐**Me** was achieved and the *Z* configuration of the C15=C16 double bond was confirmed.

**Figure 4:**
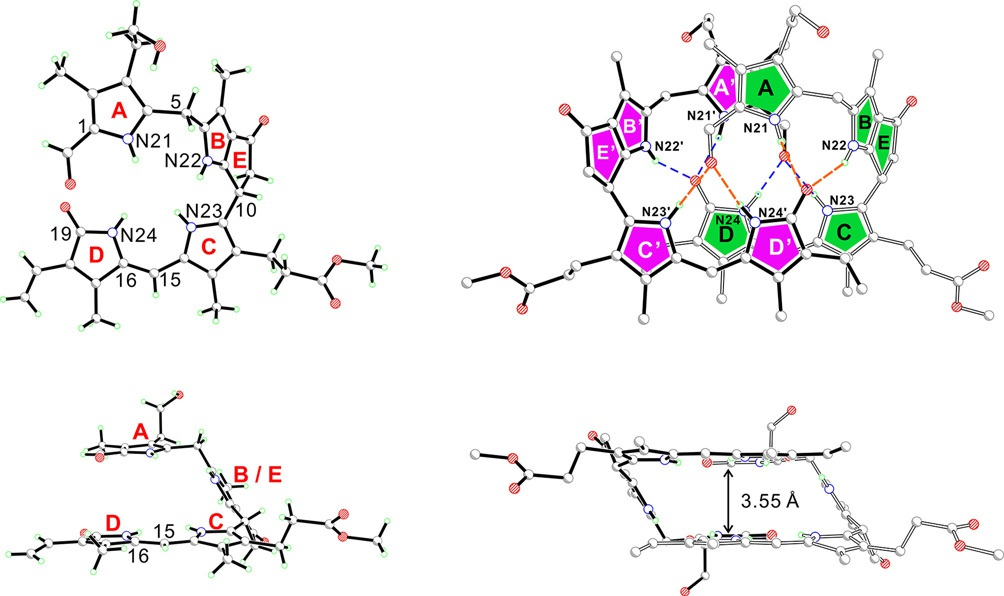
pyYCC‐Me (***Z*** **4**‐**Me**) crystallizes as a homochiral H‐bonded and π‐stacked double‐decker dimer. Left: Two projections of the crystal structure of ***Z*** **4**‐**Me** as a monomer in a ball‐stick model. Right: Two corresponding projections of the crystallographic model of the intertwined homochiral dimer (***Z*** **4**‐**Me**)_2_ highlighting its H‐bonding pattern and its π‐stacking motifs. Note: in the homodimer the C15=C16 double bonds (between rings C and D) of both monomers are positioned in close mutual proximity.

Single crystals of pyYCC‐Me ***Z*** **4**‐**Me** grew from CHCl_3_/hexane at ambient temperature. ***Z*** **4**‐**Me** crystallized in the monoclinic space group *P*−2_1_. The crystal structure of ***Z*** **4**‐**Me** confirmed its earlier NMR derived structure, including, the lactam function of ring D, as well as a Z configuration and a high double‐bond character of the C15=C16 bond (see Figure 4). The absolute configuration at C10 of pyYCC‐Me ***Z*** **4**‐**Me** was assigned on the basis of the CD spectra of ***Z*** **4**‐**Me** and YCC‐Me, the absolute structure of which has been deduced by crystallography.^31^

**Figure 5:**
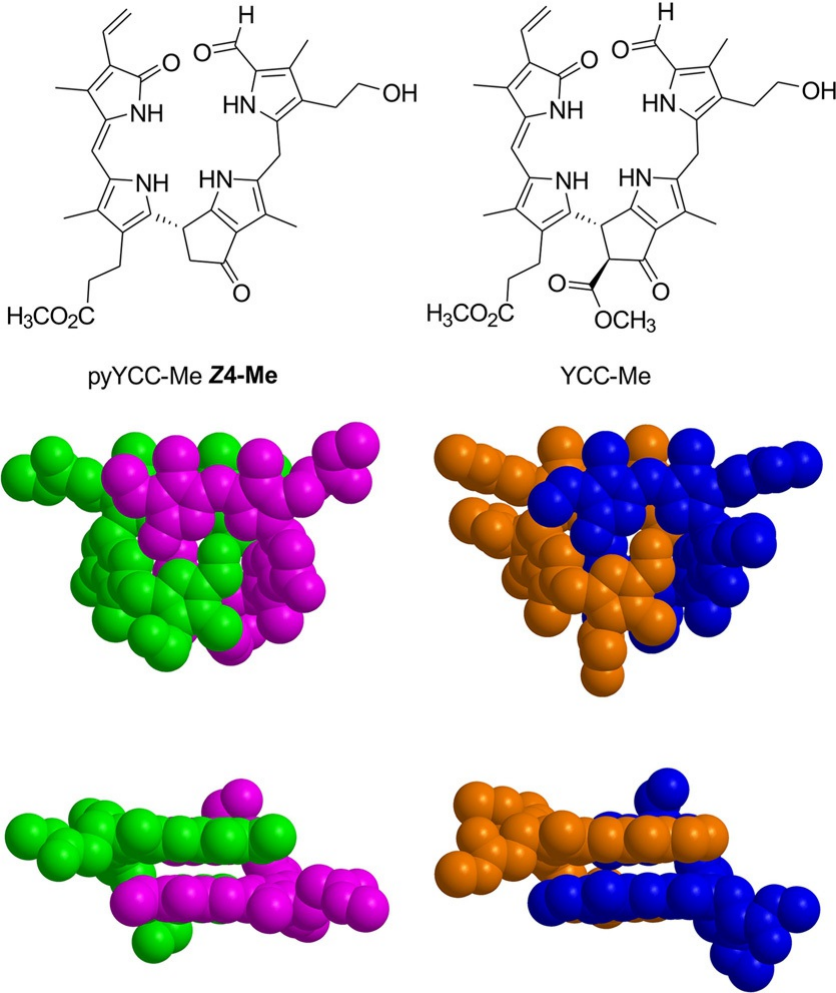
Top: Formulas of pyYCC‐Me ***Z*** **4**‐**Me** and of YCC‐Me (as monomers). Bottom: Space filling models of the crystal structures of the corresponding hydrogen‐bonded and π–π‐stacked intertwined homodimers (***Z*** **4**‐**Me**)_**2**_ (left) and (**YCC**‐**Me**)_**2**_ (right).

In the crystal, two molecules of pyYCC‐Me (***Z*** **4**‐**Me**) form a hydrogen‐bonded and π‐stacked homodimer in a “double‐decker” arrangement, separated by the ring B/E‐section as a spacer, as recently found for YCC‐Me (see Figure 5).^31^ In these C2‐symmetric dimers, the short (C15=C16) bonds of two ***Z*** **4**‐**Me** molecules are positioned nearly on top of each other. The (“bilirubin”‐type) ring C/D moiety of ***Z*** **4**‐**Me** is nearly planar, with a mean deviation of 0.056 Å, similar to the situation in YCC‐Me (0.041 Å).^31^ Likewise, the dihedral angles between the nearly coplanar rings C and D of ***Z*** **4**‐**Me** (9.1°) and of YCC‐Me (6.2°) were similarly small. The dihedral angle between rings E and C/D was 57.5° in pyYCC‐Me (***Z*** **4**‐**Me**), whereas it was 73.3° in the case of YCC‐Me. Correspondingly, the dihedral angle between ring B and the ring C/D moiety was 57.2 and 67.4° for ***Z*** **4**‐**Me** and YCC‐Me, respectively. However, rings B and E form a nearly perfect plane, with dihedral angles between their best planes of 3.5 and of 6.7°, respectively, for ***Z*** **4**‐**Me** and YCC‐Me. In ***Z*** **4**‐**Me** C10 is moved below the plane of the other ring E carbon atoms (C8, C8^1^, C8^2^, and C9) by 0.306 Å, compared to 0.361 Å in YCC‐Me. Carbon C8^2^ is positioned 0.304 Å above the plane of the other carbon atoms of ring E (C8, C8^1^, C9, and C10) in ***Z*** **4**‐**Me** versus 0.335 Å for that in YCC‐Me. Hence ring E of ***Z*** **4**‐**Me** is twisted around the bond between the sp^3^‐hybridized carbon atoms C82 and C10, but slightly less than in YCC‐Me, a likely consequence of its methoxycarbonyl group at C8^2^.

In the hydrogen‐bonded dimer structure, the ring C/D and ring C′/D′ moieties are situated above each other in nearly parallel planes (with a dihedral angle of 5.9° for pyYCC‐Me and 1.1° for YCC‐Me). The mean deviation for the more extended planes of ring C/D/A′ is 0.061 Å for pyYCC‐Me (***Z*** **4**‐**Me**), whereas that for the plane of ring C′/D′/A in YCC‐Me is 0.040 Å. The intermolecular distances for C15‐C16′ and C15′‐C16 of ***Z*** **4**‐**Me** are 3.97 Å, that is, approximately 0.3 Å longer than that in YCC‐Me.^31^ Likewise, the distance between the two parallel planes is 3.55 Å in ***Z*** **4**‐**Me**, that is, also slightly longer than in YCC‐Me, where it amounts to 3.47 Å.^31^ Hence, intermodular packing in (YCC‐Me)_2_, the dimer of the more highly substituted YCC‐Me, is remarkably more tight than in the dimer (***Z*** **4**‐**Me**)_2_ of the pyro‐YCC analogue ***Z*** **4**‐**Me**.

pyYCC‐Me (***Z*** **4**‐**Me**) and its analogue with the C8^2^ ester group, YCC‐Me, are remarkably similarly structured both in crystal (see above, Figure 5) and in solution. Indeed, ^1^H NMR spectra of both compounds overlap to a large extent, when in the same solvent, for example, in [D_6_]DMSO or in CDCl_3_. In [D_6_]DMSO pyYCC‐Me (***Z*** **4‐Me**) displays NMR spectral features of a monomeric pyro‐phyllobilin. In contrast, ^1^H NMR data of solutions of ***Z*** **4**‐**Me** in CDCl_3_, were not compatible with a monomeric structure (see Figure 3). When compared with the corresponding signals in [D_6_]DMSO, the signals of four NH groups in CDCl_3_ shift to lower field with Δ*δ* in the range of *δ*=0.50 to 1.10 ppm, that is, Δ*δ* (HN21): *δ*=1.06 ppm, Δ*δ* (HN22): *δ*=0.5 ppm, Δ*δ* (HN23): *δ*=0.65 ppm, and Δ*δ* (HN24): *δ*=1.1 ppm. Thus, the data of ***Z*** **4**‐**Me** in CDCl_3_ indicate the existence of H‐bonded dimers with intermolecular H‐bonds between O and HN functionalities, as found in the crystal. High‐field shifts of the CHO signal (Δ*δ*=−2.13 ppm) and for CH, *trans*‐H_A_C18^2^ and *cis*‐H_B_C18^2^ (of *δ*=0.58, 0.44, and 0.23 ppm, respectively) of the vinyl group in CDCl_3_ may be ascribed to shielding‐effects of the pyrrole ring A or of the conjugated ring C/D framework, respectively, on protons of the partner molecule. The solvent effects in ^1^H NMR spectra of ***Z*** **4**‐**Me** and of YCC‐Me^31^ in [D_6_]DMSO or in CDCl_3_ are similar and compatible with H‐bonded double‐decker dimer structures in CDCl_3_ solution and in the crystal. The vinyl group protons H_A_C18^2^ and H_B_C18^2^ correlate with HN23 or H_2_C1712^1^ and H_2_C12^2^, respectively, in the ^1^H,^1^H‐ROESY spectra. Such correlations could hardly occur via intramodular couplings, in view of the long distance from the vinyl group at C18 to rings B and C of a monomer. Likewise, the signal of CHO showed correlations with all NH signals, as well as a weak correlation to H_A_C8^2^. These NOE data are consistent with a solution dimer of ***Z*** **4**‐**Me**, as seen in the crystal. In fact, as for YCC‐Me, the crystal structure of pyYCC‐Me (***Z*** **4**‐**Me**) helped to rationalize the NMR spectral features of pyYCC‐Me (and pyYCC) in CDCl_3_, and the ^1^H,^1^H‐correlations fit well with the dimer structure of ***Z*** **4**‐**Me**. Therefore, hydrogen‐bonded dimers (***Z*** **4**‐**Me**)_2_ also predominate in CDCl_3_, as deduced earlier for the analogue YCC‐Me.^31^

**Figure 6:**
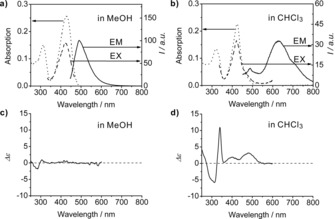
Solvent‐dependent UV/Vis, fluorescence, and CD spectra of ***Z*** **4**‐**Me**. a, b) UV/Vis‐spectra (dotted line), fluorescence excitation (EX, dashed line), and emission (EM, solid line) spectra of ***Z*** **4**‐**Me**. a) A 6.0×10^−6^ m solution of ***Z*** **4**‐**Me** in MeOH exhibits spectral features of the monomer. b) A 5.2×10^−6^ m solution of ***Z*** **4**‐**Me** in CHCl_3_ features excimer luminescence due to the presence of hydrogen‐bonded dimers. c, d) CD spectra of the solutions of ***Z*** **4**‐**Me** in MeOH (left) and in CHCl_3_ (right), which display the spectral features of monomers and of a *P*‐helical hydrogen‐bonded dimer, respectively.

Depending upon the solvent and its polarity, pyYCC‐Me (***Z*** **4**‐**Me**) predominantly exists in solution as a monomer or as the hydrogen‐bonded dimer (***Z*** **4**‐**Me**)_2_. UV/Vis, fluorescence and CD spectra reflected this fact consistently (see Figure 6), as did the observed dual path photochemistry of YCC‐Me.^31^ UV/Vis‐spectra of ***Z*** **4**‐**Me** were solvent dependent, and they were slightly different in MeOH and in CHCl_3_. An absorption “tail” is seen at low transition energies in the latter solvent (see the Supporting Information, Figure S4). Likewise, CD spectra of ***Z*** **4**‐**Me** are also strongly medium‐responsive, as observed earlier for YCC‐Me.^31^ Solutions of ***Z*** **4**‐**Me** in MeOH exhibit weak CD‐effects near 340 nm. In contrast, they display a strong and “positive” Cotton‐effect near 340 nm in CHCl_3_, diagnostic of a P‐type arrangement of rings A of hydrogen‐bonded dimer (***Z*** **4**‐**Me**)_2_, as found in the crystal.

Fluorescence spectra of ***Z*** **4**‐**Me** were also strongly solvent dependent. In polar solvents at 23 °C, the fluorescence of ***Z*** **4**‐**Me** was weak, characteristic of a deactivation of the excited singlet state by a rapid twist and eventual *Z*/*E*‐isomerization around a critical (C=C) bond. The fluorescence emission in MeOH had a pronounced maximum near 490 nm, but near 630 nm in CHCl_3_. The former situation suggests emission from the monomeric state of ***Z*** **4**‐**Me**, the latter excimer‐emission from the preformed noncovalent dimer (***Z*** **4**‐**Me**)_2_.

**Figure 7:**
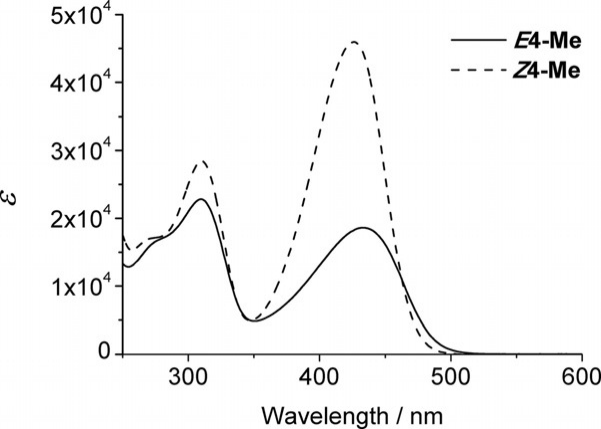
UV/Vis spectra of ***E*** **4**‐**Me** (4.8×10^−4^ m, solid line) and of ***Z*** **4**‐**Me** (1.7×10^−4^ m, dashed line) in MeOH.

Irradiation of a solution of pyYCC‐Me (***Z*** **4**‐**Me**) in MeOH with the light of the fluorescent lamp caused efficient conversion to the *E* isomer ***E*** **4**‐**Me** reaching a steady state near 50 % conversion. The photochemically produced *E* isomer ***E*** **4**‐**Me** was fully characterized spectroscopically (see the Supporting Information, Figures S7–S9). In particular, *Z* to *E* isomerization is reflected by changes of the relative absorption intensities in the UV‐ and Vis‐regions of the UV/Vis‐spectra of ***E*** **4**‐**Me** compared to that of ***Z*** **4**‐**Me** (see Figure 7), consistent with the earlier observations made with the corresponding *E*/*Z*‐isomeric versions of YCC.

**Figure 8:**
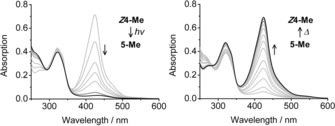
Left: Photodimerization of yellow ***Z*** **4**‐**Me** in CHCl_3_ (1.78×10^−4^ m) at 0 °C furnishing colorless **5**‐**Me**. Right: Thermolysis of **5**‐**Me** (8.9×10^−5^ m) at 50 °C regenerates ***Z*** **4**‐**Me**.

**Scheme 6:**
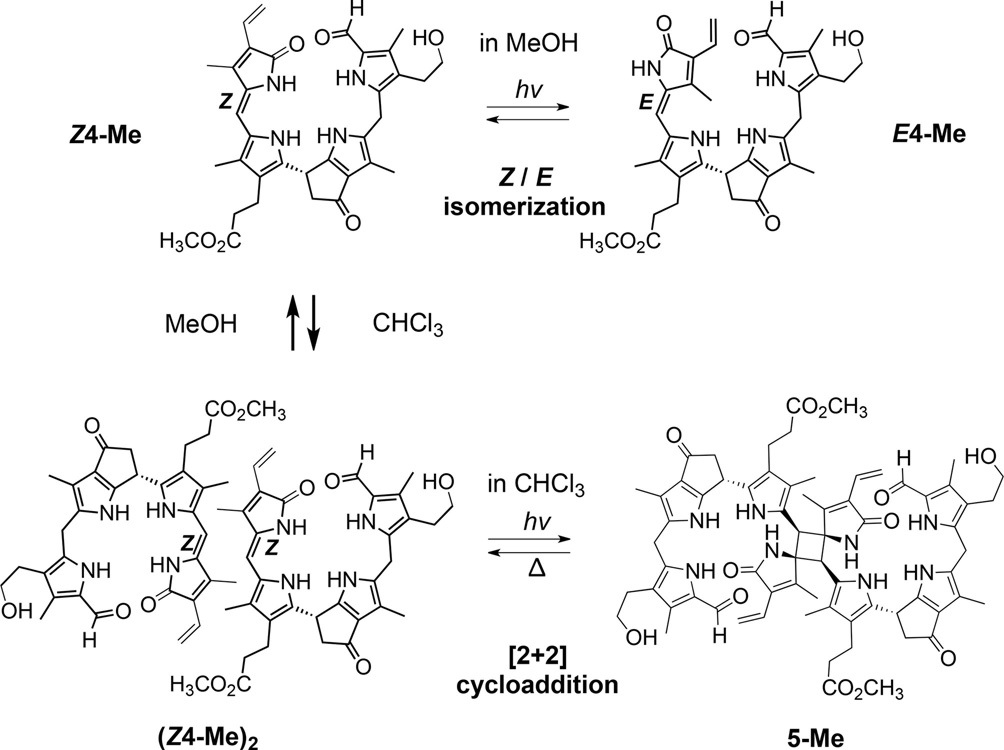
Semisynthetic pyro‐type phylloxanthobilin ***Z*** **4**‐**Me** behaves as a medium responsive photoswitch. Photoexcited pyYCC *Z*‐ and *E* isomers interconvert reversibly in the polar solvent MeOH. In apolar solvents, like CHCl_3_, ***Z*** **4**‐**Me** preorganizes to (***Z*** **4**‐**Me**)_2_ by hydrogen‐bonded self‐assembly. (***Z*** **4**‐**Me**)_2_ undergoes clean [2+2]‐photocycloaddition to the *C*_2_‐symmetric octapyrrole **5‐Me**, which reverts to (***Z*** **4**‐**Me**)_**2**_ in the dark after thermal activation.

**Figure 9:**
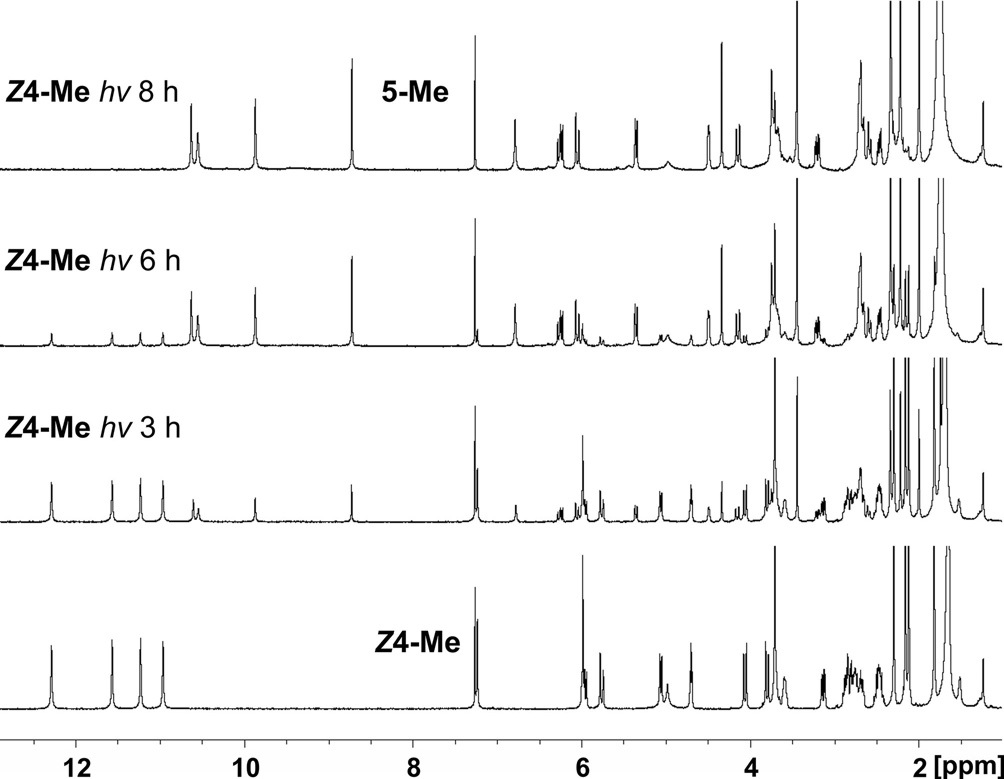
NMR‐analysis of the photodimerization of ***Z*** **4**‐**Me** (1.1×10^−2^ m) to **5**‐**Me** after irradiation by fluorescent lamp for 3, 6, and 8 h at 0 °C. The change in the ^1^H NMR indicated the complete conversion of ***Z*** **4**‐**Me** to the octapyrrole **5**‐**Me** after 8 h irradiation by the fluorescence lamp.

In marked contrast to the situation in MeOH, exposure of a solution of ***Z*** **4**‐**Me** in CDCl_3_ to the light of the fluorescent lamp at 0 °C caused bleaching of the characteristic 420 nm absorption (see Figure 8) and resulted in clean, regio‐ and stereoselective conversion to the colorless octapyrrolic photodimer **5**‐**Me** (>95 %), as derived from extensive NMR analysis (see Scheme 6 and Figure 9).

The covalent C_2_‐symmetric [2+2]‐photodimer **5**‐**Me** was rather stable at 0 °C and its structure could be thoroughly investigated, based on homo‐ and heteronuclear NMR spectra. The two halves of the octapyrrole **5**‐**Me** are connected via a cyclobutane core, characterized by methine resonances at *δ*=4.33 ppm (^1^H) and at *δ*=43.5 ppm (^13^C), as well as by a ^13^C‐signal of the quaternary carbon center at *δ*=73.2 ppm. The hydrogen‐bonding contacts seen in the crystal and in CDCl_3_ solution of the homodimer (***Z*** **4**‐**Me**)_2_ appear to be kept in **5**‐**Me**. The UV/Vis‐spectrum of the dimer **5**‐**Me** exhibited an absorption maximum near 320 nm (due to its formyl–pyrrole unit), and is consistent with the interruption of the main chromophore of ***Z*** **4**‐**Me** at the C15‐mesoposition. The strong Cotton effect near 330 nm in the CD‐spectrum of **5**‐**Me** (see Figure S13, Supporting Information) suggested a similar P‐type arrangement of the rings A/A′ of **5**‐**Me**, as shown in the crystal for the structure of the H‐bonded dimer (***Z*** **4**‐**Me**)_**2**_.

**Figure 10:**
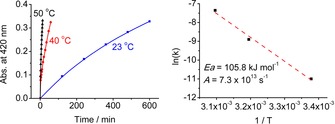
Kinetic analysis of the thermally induced [2+2]‐cycloreversion of **5**‐**Me** (8.9×10^−5^ m) to (***Z*** **4**‐**Me**)_**2**_ in Ar‐purged acid‐free CHCl_3_ in the dark. Left: Formation of ***Z*** **4**‐**Me** monitored at the indicated temperatures via the absorption near 420 nm. Right: Arrhenius values *E*_a_ and A from the linear fit of the reaction rates *k* determined at the temperatures of 23, 40, and 50 °C.

Thermolytic [2+2]‐cycloreversion of the thermally labile covalent dimer **5**‐**Me** occurred slowly at room temperature in CDCl_3_ and furnished the hydrogen‐bonded dimer (***Z*** **4**‐**Me**)_**2**_ (see Scheme 6 and Figure 10). In acid‐free CHCl_3_ the decomposition of the colorless, covalent photodimer **5**‐**Me** displayed first‐order kinetics with a half‐life of about 18 min at 50 °C and about 700 min at 23 °C. Using the Eyring equation to analyze the decomposition kinetics, enthalpy and entropy of activation were calculated as 103.2 kJ mol^−1^ and 11.9 J K mol^−1^, respectively. From a corresponding Arrhenius analysis, the activation energy of this ring‐opening reaction was determined as 105.8 kJ mol^−1^ and a remarkably high frequency factor (7.3×10^13^ s) was calculated (see Figure 10 and the Supporting Information, Figures S14 and S15). Hence, opening of the cyclobutane ring of **5**‐**Me** in CHCl_3_ was more rapid by about eight times than that observed for the cycloreversion of the corresponding photodimer of YCC‐Me on the basis of the half‐life at 50 °C.^31^

**Figure 11:**
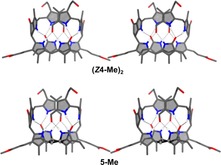
Stereoprojection of the quantum chemically optimized structures (***Z*** **4**‐**Me**)_**2**_ and **5**‐**Me**. Note: upon [2+2] cycloaddition of (***Z*** **4**‐**Me**)_**2**_ to form **5**‐**Me** the H‐bonded network is kept intact (dashed lines: H‐bonds; color code: C gray, O red, N blue, H white; see Scheme 6 for the chemical formulas of (***Z*** **4**‐**Me**)_2_ and **5**‐**Me**)).

The structures of the monomeric pyro‐type phylloxanthobilins ***E*** **4**‐**Me** and ***Z*** **4**‐**Me** (see the Supporting Information, Figure S16), as well as of the hydrogen‐bonded π‐stacked homodimer (***Z*** **4**‐**Me**)_**2**_ and the covalent photodimer **5**‐**Me** (see Figure 11 and the Supporting Information, Table S5) were modeled with density functional theory (BP86/def2‐TZVP/D3; for details see the Experimental Section).

The optimized structures of the monomers ***E*** **4**‐**Me** and ***Z*** **4**‐**Me** were calculated (see their stereo projections in the Supporting Information, Figure S16), reproducing the higher stability of ***Z*** **4**‐**Me** compared to ***E*** **4**‐**Me** (Δ*G*=−18.7 kJ mol^−1^). Both monomers have—in their lowest‐energy conformation in the gas phase—two intramolecular hydrogen bonds, resulting in ‘tucked‐in“ conformations. Among the H‐bonds is a common one between the nitrogen of ring B and the carbonyl oxygen atom of the methyl ester substituent in ring C. The second H‐bond is formed between the terminal OH‐group at the ethyl‐substituent of ring A and, for ***Z*** **4**‐**Me**, the NH of ring C, or, for ***E*** **4**‐**Me**, the lactam carbonyl oxygen atom of ring D. In the case of ***E*** **4**‐**Me**, the calculated ”tucked‐in“ H‐bonded conformation helps explain qualitatively the noteworthy observed ^1^H,^1^H‐NOE‐correlations (in CDCl_3_) between the ”terminal“ rings A and D (see Figures 4 and the Supporting Information, Figure S1 for atom numbering and ring nomenclature).

Stereoprojections of calculated structures of (***Z*** **4**‐**Me**)_**2**_ and **5**‐**Me** are depicted in Figure 11. Quantum chemical calculations confirmed the integrity of the H‐bonded network in **5**‐**Me** as found for the previously reported **2**‐**Me**. The computed structural models of pyYCC‐Me (***Z*** **4**‐**Me**)_**2**_ and of **5**‐**Me** featured intramolecular (inter‐ and intramodular) distances that were consistent with the observed NOEs and with the proposed network of eight hydrogen bonds that appears to be conserved during the interconversion of (***Z*** **4**‐**Me**)_**2**_ and **5**‐**Me**. The optimized structures of the homodimer pairs (***Z*** **4**‐**Me**)_**2**_ and (YCC‐Me)_**2**_, as well as the main framework of the corresponding photodimer pairs **5**‐**Me** and **2**‐**Me** are calculated to be highly similar (Supporting Information, Figures S17 and S18).

The cyclobutane unit of **5**‐**Me** shows remarkable differences in the calculated C−C bond lengths and the newly formed C15−C16′ and C16−C15′ are 0.08 Å longer than C15−C16 and C16′−C15′. This effect is more pronounced for **5**‐**Me** than was found for **2**‐**Me**. Concerning the π–π stacking of the α‐formyl pyrrole rings A, the intermodular distances between these rings are little affected by the formation of the cyclobutane ring in **5**‐**Me** and are very similar as in the H‐bonded dimer (***Z*** **4**‐**Me**)_**2**_. Whereas the crystallographic dihedral angle between the ring B and C/D moieties is 57.2 (***Z*** **4**‐**Me**)_**2**_ and 67.4° for (YCC‐Me)_2_, quantum chemical calculations show a remarkably opposite trend. Hence, the B‐C/D dihedral, 64.4°, is calculated to be larger for (***Z*** **4**‐**Me**)_**2**_ than for (YCC‐Me)_2_ (58.4°). These differences between quantum chemical calculations and X‐ray crystal structures may be attributed to packing effects. Likewise, the photodimer **5**‐**Me** (62.1°) exhibits a larger B‐C/D dihedral angle than in the analogue **2**‐**Me** (56.0°), consistent with a more rectangular shape of the cyclobutane moiety in the less substituted species.

## Conclusions

This paper introduces the pyNCC **3** as a representative of the pyro‐phyllobilins, so far unknown PB analogues, which lack the carboxylate function at their ring E moiety. Typical NCCs carry a β‐keto carboxylate grouping at ring E, which is inherited from their Chl precursors, and which renders their C8^2^ asymmetric and prone to solvent‐assisted loss of a proton.^5^, ^6^ Hence, natural NCCs (as well as YCCs and Chls) exist as C8^2^‐epimeric pairs exhibiting significantly different relative stabilities.^28^, ^31^, ^32^ However, as shown here, the carboxylate substituent at C8^2^ has little effect on the flexible structure of NCCs and on the photoreactivity of YCCs. Thus, pyPBs are close biosimilars of the abundant natural PBs, and it may be rewarding, indeed, to search routinely for pyNCCs in extracts of plant materials.

Spontaneous loss of carbon dioxide from 8^2^‐carboxy‐NCCs (such as **2**) is remarkably slow.^25^ This behavior of **2** and of related polar NCCs^16^, ^17^, ^25^ contrasts with the situation with 13^2^‐carboxy‐pyro‐pheophorbide *a* 33 and known corresponding RCC forms,^24^ in which decarboxylation at C8^2^ to pyPheo *a* and pyRCC, respectively (see Scheme 2), occurs easily and is activated by extensive conjugative stabilization of the critical decarboxylation intermediates. Along these lines, the origin of remarkable Chl‐derived linear tetrapyrroles that lack a carboxylate group at their rings E is also intriguing, which represent the bioluminescent emitter in Krill (*Euphausia pacifica*)^34^ and luciferin of dinoflagellates.^35^ These bilin‐type natural products may be derived from a pyro‐pheophorbide precursor or from decarboxylation of an unknown later breakdown intermediate.

The novel pyro‐type YCC (pyYCC), as well as YCCs, the bright yellow bilin‐type products of Chl breakdown in higher plants, feature the basic chromophore of the heme‐breakdown product bilirubin (BR).^7^, ^36^, ^37^ Hence, YCCs and pyYCCs could be considered PB‐type analogues of the yellow bile pigment BR.^6^ Whereas a crystal structure of BR revealed it in an intramolecular H‐bonded monomeric form with a “roof tile” shape,^37^ pyYCC‐Me, the natural phylloxanthobilin YCC and its methyl ester YCC‐Me assemble into self‐templated dimeric “double‐decker” structures, spanned by the characteristic B/E‐ring moiety of the PBs. The C2‐symmetric dimers constitute a “hand shake” motif,^31^ which does not depend significantly on the presence of a carboxylate function (as in YCC or YCC‐Me), or its absence in pyYCC (and in pyYCC‐Me), as shown here. Indeed, the lack of a carboxylate substituent at the C8^2^‐position in pyYCC‐Me (***Z*** **4**‐**Me**) has only a small effect on the whole structure, most clearly seen in the crystal, in which only slightly differing dihedral angles between the planes of the ring B/E and ring C/D subsystems are observed. Aside of this, the substituents at C8^2^ show negligible effects on the structure, the chemical reactivity and, photochemistry of both types of yellow phyllobilins.

pyYCC‐Me (***Z*** **4**‐**Me**) represents a medium‐responsive photoswitch similar to YCC (and YCC‐Me).^31^ In polar solution, in which YCCs and pyYCCs prefer to be monomeric, they display insignificant luminescence and undergo clean photochemical *Z*/*E*‐isomerization. Hence, in such solvents, YCCs and pyYCC show photochemistry remarkably comparable to that of the natural heme catabolite BR.^37^ BR undergoes a related, but modestly selective, photoinduced *Z* to *E* isomerization, which is considered the basis of the blue‐light therapy of neonatal jaundice.^36^ However, the photochemistry of YCCs and pyYCCs is strongly medium responsive, and in apolar media, the self‐assembled *Z* isomers YCC, YCC‐Me, and pyYCC‐Me (***Z*** **4**‐**Me**) undergo a clean and surprisingly efficient photoinduced [2+2]‐cycloaddition to homodimeric octapyrroles, such as **5**‐**Me**.

The novel, “non‐natural” colorless pyNCC **3** and the yellow pyYCC **4** may be considered to represent close biosimilars of NCCs and YCCs, their known natural relatives. By possessing a unique ring E moiety, all phyllobilins are conformationally restricted bilin‐type tetrapyrroles. Hence, their overall structures and their chemical properties deviate systematically from those of the related heme‐derived bilins, which play a range of important biological roles:^7^, ^37^, ^38^, ^39^, ^40^ For example, phytochrome with bound phytochromobilin (PΦB), when excited by visible light, undergoes fast *Z*/*E*‐isomerization of the C15=C16 bond.^39^ This property of this natural bilin‐type photoswitch is used in higher plants for their crucial photoregulation.^40^ YCC and related yellow phyllobilins also undergo efficient photoinduced *Z*/*E*‐isomerization of their C15=C16 bond in polar solutions. Colored phyllobilins (collectively named phyllochromobilins), such as YCCs and PiCCs (pink oxidation products of YCCs), are also excellent ligands for some transition‐metal ions and their metal complexes display a further spectrum of colors and (photo)chemistry.^[41**]**^ Furthermore, persistent blue fluorescent FCCs, classified as “hypermodified” FCCs (*hm*FCCs), occur in some fruit and leaves of evergreens (such as bananas), acting as optical brighteners in them.^42^ Clearly, the interesting antioxidant properties of some PBs,^43^ their bright colors and their photochemistry^29^, ^31^ constitute a range of chemical qualities that may be essential for the still elusive biological roles of PBs in fruit and leaves.^6^ As pyro‐phyllobilins (pyPBs) appear to feature similar structures and (photo)chemistry as the corresponding natural PBs, they could, indeed, be seen as biosimilars with a related potential for active or inhibitory participation in physiological processes.

## Experimental Section

### General

CHCl_3_, *n*‐hexane reagent‐grade commercial; MeOH (HPLC grade) and CH_3_CN (HPLC grade), from VMR (Leuven, Belgium); water, from Millipore S.A.S. Milli‐Q Academic system (18.2 MΩ cm^−1^, Molsheim, France); ACS reagent KH_2_PO_4_ and K_2_HPO_4_, acetic acid (AcOH), and trifluoroacetic acid (TFA) from Sigma–Aldrich (Steinheim, Germany). NCC **1** was isolated from senescent leaves of *Cercidiphyllum japonicum*, as reported.^27^, ^29^ Sep‐Pak‐C18 Cartridges, from Waters Associates (Milford, USA); reverse‐phase silica gel (Sepra C18‐E, 50 μm, 65 Å), from Phenomenex (Aschaffenburg, Germany). pH‐values, measured with a WTW Sentix 21 electrode, WTW pH535 digital pH meter. Light source for photochemistry: fluorescence lamp (21 W), from Sylvania FHE 21W/T5/830 (London, England).

### Spectroscopy

UV/Vis: Varian Cary 60 spectrophotometer; *λ*_max_ in nm (log *ϵ*). CD‐spectra: JASCO J‐715 spectropolarimeter; *λ*_max_ and *λ*_min_ in nm (Δ*ϵ*). ^1^H NMR: Varian UNITY plus 500; *δ* in ppm with *δ*(CHD_2_OD)=3.31 ppm, *δ*(CHCl_3_)=7.26 ppm, *δ*(CD_3_SOCD_2_H)=2.50 ppm, coupling constant *J*_HH_ in Hz; ^13^C‐NMR: chemical shift values and signal assignments from ^1^H,^13^C‐HSQC and ^1^H,^13^C‐HMBC spectra. ESI‐MS: Finnigan LCQ Classic, ESI source, positive‐ion mode, flow rate 2 mL min^−1^, solvent water/MeOH. FAB‐MS: Finnigan MAT‐95, positive‐ion mode, glycerin matrix; *m*/*z* (rel. intensity %). Analytical HPLC: Gynkotek “high‐precision pump” 480 G with vacuum online degasser, Gynkotek diode array detector DA340, all chromatograms were taken at room temperature, Phenomenex, ODS‐Hypersil 5 μ, 250×4.6 mm i.d. precolumn was used with a flow rate: 0.5 mL min^−1^; solvent A: 50 mm aq. potassium phosphate (pH 7.0), solvent B: MeOH, solvent C: H_2_O; solvent composition (A/B/C): 0–5 min: 60/40/0; 5–15 min: from 60/40/0 to 30/70/0; 15–25 min: from 30/70/0 to 0/100/0; 25–35 min: from 0/100/0; 35–37 min: 0/100/0 to 0/90/10; 37–42 min: from 0/90/10 to 60/40/0.

### Synthesis of the NCC 2 by ester hydrolysis of the NCC 1

NCC **1** (200 mg, 310 μmol) and porcine liver esterase (25 mg, 163 U mg^−1^) were dissolved in 50 mL potassium phosphate buffer (pH 7.9, 0.25 m). The reaction mixture was stirred at 39 °C under Ar in the dark. After 5 days, the reaction mixture was diluted with 15 mL of MeOH and then loaded on a Sep‐Pak cartridge. The polar NCC **2** was eluted with MeOH, the eluate was frozen by liquid N_2_ and vacuum lyophilized to give 188 mg NCC **2** (96 % yield). The NCC **2** was analyzed and its structure was deduced, as described below, confirming its identity with *So*‐NCC‐3 from senescent leaves of spinach.^25^

**Spectroanalytical data of NCC 2**: UV/Vis (9.8×10^−5^ mol L^−1^, H_2_O): *λ*_max_ (log *ϵ*) 208 (4.55), 242sh (4.27), 316 (4.23); CD: (1.3× 10^−4^ mol L^−1^, MeOH): *λ*_min/max_, (Δ*ϵ*) 205 (−10.1), 227 (16.9), 253sh (2.4), 314 nm (0.6); ^1^H NMR (500 MHz, CD_3_OD, 26 °C): *δ*=1.92 (s, H_3_C13^1^), 1.94 (s, H_3_C17^1^), 2.10 (s, H_3_C7^1^), 2.25 (m, H_A_C12^2^), 2.26 (s, H_3_C2^1^), 2.35 (m, H_B_C12^2^), 2.52 (dd, *J=*8.9/14.3, H_A_C15), 2.62 (m, H_2_C3^1^) superimposed by H_2_C12^1^, 2.85 (dd, *J=*5.5/14.3, H_B_C15), 3.48 (m, H_2_C3^2^), 3.92 (m, H_2_C5), 4.02 (dd, *J=*5.5/8.9, HC16), 4.88 (s, HC10), 5.34 (dd, *J=*2.3/11.7, H_A_C18^2^), 6.44 (dd, *J=*2.6/17.7, H_B_C18^2^), 6.44 (dd, *J=*11.3/18.7, HC18^1^), 9.34 ppm (s, HC20); FAB‐MS (glycerol matrix): *m*/*z* (%): 671.3 (20), 670.4 (33), 669.3 (63, [*M*+K]^+^); 633.3 (17), 632.3 (43), 631.3 (100, [*M*+H]^+^); 626.0 (15), 625.2 (38, [*M*−CO_2_+K]^+^); 508.2 (24, [*M*−ring A+H]^+^); 464.2 (19, [*M*−ring A−CO_2_+H]^+^); HR‐FAB‐MS: *m*/*z* (%): calcd for [*M*+H]^+^ C_34_H_39_N_4_O_8_ *=*631.277; found: 631.277±0.005.

### Synthesis of pyNCC (3) by decarboxylation of NCC 2

The polar NCC **2** (680 mg, 1.8 mmol) was dissolved in H_2_O (116 mL) under Ar with assistance of ultrasound. To the above solution, aqueous 20 mm H_2_SO_4_ was added (195 mL), and the stirred solution was purged with Ar. After 7 h at 80 °C, the reaction mixture was cooled down to room temperature and a precipitate of raw pyNCC **3** formed. The precipitate was separated off by filtration and washed with hexane. After drying, raw **3** (558 mg) was obtained. A sample of raw **3** (100 mg) was purified further by preparative HPLC. The fractions of purified **3** were combined, diluted 1:1 with H_2_O and loaded on a Sep‐Pak cartridge. After washing with H_2_O (40 mL), the product was eluted with MeOH. The solvent was removed under reduced pressure and the sample was dried under high vacuum, yielding pyNCC **3** (33 mg, 29 % yield). The fractions of a slightly more polar NCC were also collected and worked up, furnishing an NCC (8 mg, 7 % yield), characterized as *epi*‐**3** (see the following and main text).

**Spectroanalytical data of pyNCC 3**: UV/Vis (4.1×10^−4^ mol L^−1^, MeOH): *λ*_max_ (log *ϵ*) 214 (4.52), 238sh (4.37), 310 (4.32); CD: (4.1×10^−4^ mol L^−1^, MeOH): *λ*_min/max_ (Δ*ϵ*) 203 (−24.9), 227 (33.4), 251sh (−4.2), 310 nm (3.3); ^1^H NMR (500 MHz, CD_3_OD, 26 °C): *δ*=1.91 (s, H_3_C13^1^), 1.94 (s, H_3_C17^1^), 2.13 (s, H_3_C7^1^), 2.25 (s, H_3_C2^1^), 2.34 (m, H_A_C12^2^), 2.40 (m, H_B_C12^2^), 2.56 (dd, *J=*8.8/14.6, H_A_C15), 2.62 (t, *J=*7.3, H_2_C3^1^), 2.68 (m, H_2_C12^1^), 2.72 (m, H_A_C8^2^), 2.83 (dd, *J=*4.9/14.6, H_B_C15), 3.27 (dd, *J=*6.8/18.6, H_B_C8^2^), 3.50 (m, H_2_C3^2^), 3.91/3.95 (AB‐system, *J=*16.6, H_2_C5), 4.00 (dd, *J=*5.8/7.8, HC16), 4.58 (dd, *J=*2.0/6.8, HC10), 5.35 (dd, *J=*2.0/11.7, H_A_C18^2^), 6.09 (dd, *J=*2.0/17.6, H_B_C18^2^), 6.43 (dd, *J=*11.7/17.6, HC18^1^), 9.34 ppm (s, HC20); ^13^C‐NMR (CD_3_OD, 26 °C): *δ*=8.8 (2^1^), 9.1 (7^1^), 9.2 (13^1^), 12.4 (17^1^), 21.0 (12^1^), 23.6 (5), 27.9 (3^1^), 30.0 (15), 32.3 (10), 37.1 (12^2^), 51.8 (8^2^), 61.6 (16), 65.6 (3^2^), 112.1 (7), 115.4 (13), 118.9 (12), 119.5 (18^2^), 120.0(3), 126.3 (11 and 14), 127.4 (8 and 18^1^), 129.2 (1), 133.4 (6), 135.2 (2), 138.9 (4), 140.6 (18), 157.0 (17), 162.0 (9), 174.6 (19), 177.9 (12^3^), 200.6 ppm (8^1^); HR‐ESI‐MS *m*/*z*: calcd for C_33_H_39_N_4_O_6_: 587.2864; found: 587.2870 ([*M*+H]^+^); FAB‐MS (glycerol matrix): *m*/*z* (%): calcd for [*M*+H]^+^ C_33_H_39_N_4_O_6_: 587.3; found: 588.2 (42), 587.2 (100, [*M*+H]^+^), 464.1 (52, [*M*−ring A+H]^+^); HR‐FAB‐MS: *m*/*z*: calcd for [*M*+H]^+^ C_33_H_39_N_4_O_6_: 587.2864; found: 587.2870 [*M*+H]^+^.

**Spectroanalytical data of epimer** ***epi‐*** **3**: UV/Vis (3.3×10^−4^ mol L^−1^, MeOH): *λ*_max_ (log *ϵ*) 214 (4.53), 238sh (4.39), 310 (4.33); CD: (3.3×10^−4^ mol L^−1^, MeOH): *λ*_min/max_ (Δ*ϵ*) 206 (−16.2), 229 (20.1), 253sh (1.3), 279 (−10.4), 309 nm (3.0); ^1^H NMR (500 MHz, CD_3_OD, 26 °C): *δ*=1.87 (s, H_3_C13^1^), 1.93 (s, H_3_C17^1^), 2.11 (s, H_3_C7^1^), 2.26 (s, H_3_C2^1^), 2.36 (t, *J=*6.8, H_2_C12^2^), 2.52 (dd, *J=*8.8/14.6, H_A_C15), 2.61 (t, *J=*7.3, H_2_C3^1^), 2.70 (m, H_2_C12^1^), 2.75 (m, H_A_C8^2^), 2.80 (m, H_B_C15), 3.20 (dd, *J=*6.8/18.6, H_B_C8^2^), 3.46 (t, *J=*6.8, H_2_C3^2^), 3.92 (m, H_2_C5), 4.05 (dd, *J=*5.9/8.8, HC16), 4.63 (dd, *J=*1.9/6.8, HC10), 5.34 (d, *J=*11.7, H_A_C18^2^), 6.08 (dd, *J=*2.9/18.6, H_B_C18^2^), 6.43 (dd, *J=*11.7/18.6, HC18^1^), 9.35 ppm (s, HC20); FAB‐MS (glycerol matrix): *m*/*z* (%): calcd for [*M*+H]^+^ C_33_H_39_N_4_O_6_: 587.3, 464.1 (36, [*M*−ring A+H]^+^); found: 588.2 (46), 587.2 (100, [*M*+H]^+^).

### Synthesis of pyYCC 4 by oxidation of pyNCC 3

A freshly ground slurry of green/yellow *Spathiphyllum wallisii* leaves (*Sw*‐leaves, about 25 cm^2^ leaf area) was added to a solution of pyNCC **3** (8.2 mg, 14 μmol) in a 1:1 mixture of MeOH/pH 5.2 buffer (6 mL). The above suspension was stirred for 22 h at 23 °C under 1 atm. of O_2_ in the dark. The reaction mixture was filtered through a Celite pad. The filtrate was washed with *n*‐hexane (4×10 mL) and diluted with aqueous AcOH (∼80 mL, 5.5 %, pH 2.0). After 4 h stirring under N_2_, the reaction mixture was loaded on a Sep‐Pak cartridge. Raw yellow fractions of the pyYCC **4** were eluted with MeOH/potassium phosphate buffer (pH 7.0, 50 mm) (55/45, v/v). The obtained filtrate was combined, diluted 1:1 with H_2_O, and loaded on a Sep‐Pak cartridge. After washing with 100 mL H_2_O, the product was eluted with MeOH. After removing most of the MeOH under reduced pressure, the sample of pyYCC **4** was obtained by lyophilization. PyYCC **4** was obtained as a yellow powder (4.8 mg, 59 % yield).

**Spectroanalytical data of pyYCC 4**: UV/Vis (1.4×10^−5^ mol L^−1^, MeOH): *λ*_max_ (log *ϵ*) 274 (4.33), 310 (4.48), 427 (4.63); CD: (1.4×10^−5^ mol L^−1^, MeOH): *λ*_min/max_ (Δ*ϵ*) 278 (−3.21), 306 (1.1), 419 nm (1.1); ^1^H NMR (500 MHz, CD_3_OD, 25 °C): *δ*=2.13 (s, H_3_C7^1^), 2.15 (s, H_3_C13^1^), 2.19 (s, H_3_C17^1^), 2.21 (s, H_3_C2^1^), 2.35 (t, *J=*7.3, H_2_C12^2^), 2.61 (td, *J=*2.2/7.4, H_2_C3^1^), 2.71 (m, H_A_C12^1^), 2.80 (m, H_B_C12^1^), 2.89 (dd, *J=*2.9/17.8, H_A_C8^2^), 3.24 (dd, *J=*7.1/17.8, H_B_C8^2^), 3.44 (t, *J=*7.4, H_2_C3^2^), 3.91/3.94 (AB system, *J=*16.8, H_2_C5), 4.75 (dd, *J=*2.9/7.1, HC10), 5.32 (dd, *J=*2.2/11.7, H_A_C18^2^), 6.09 (dd, *J=*2.2/17.7, H_B_C18^2^), 6.21 (s, HC15), 6.54 (dd, *J=*11.7/17.7, HC18^1^), 9.24 ppm (s, HC20);^13^C NMR (CD_3_OD, 25 °C): *δ*=8.8 (2^1^), 9.4 (7^1^ and 17^1^), 9.6 (13^1^), 22.0 (12^1^), 22.6 (5), 27.9 (3^1^), 32.4 (10), 40.1 (12^2^), 50.5 (8^2^), 62.6 (3^2^), 102.8 (15), 111.6 (7), 117.8 (18^2^), 120.5 (3), 123.2 (12), 124.4 (18), 125.5 (14), 125.8 (13), 127.0 (8), 127.3 (18^1^), 127.4 (1), 130.1 (16), 133.7 (6 and 11), 134.5 (2), 138.8 (4), 143.3 (17), 173.0 (19), 182.0 (12^3^), 198.9 ppm (8^1^); ESI‐MS *m*/*z* (%):calcd for [*M*+H]^+^ C_33_H_37_N_4_O_6_: 585.3; found: 1127.0 (11, [2*M* <m+>K]^+^), 1191.1 (15, [2*m*+Na]^+^), 1169.0 (10, [2*m*+H]^+^), 625.3 (71), 624.3 (30), 623.3 (15, [*M*+K]^+^), 609.3 (12), 608.3 (38), 607.3 (100, [*M*+Na]^+^), 587.3 (5), 586.3 (16), 585.3 (42, [*M*+H]^+^).

### Synthesis of *Z*4**‐**Me from 4

Compound **4** (2.7 mg, 4.6 μmol) and BOP (4 mg, 9.0 μmol) were dissolved in dry MeOH (2 mL). TEA (5 μL) was added at 23 °C to the above solution. The reaction mixture was stirred at room temperature overnight under N_2_, and diluted subsequently with potassium phosphate buffer (10 mL, pH 7.0, 50 mm). The yellow pyYCC methyl ester ***Z*** **4**‐**Me** was extracted by CH_2_Cl_2_ (3×10 mL). The organic phase was filtered through a plug of dry cotton wool and evaporated to dryness under reduced pressure. The dark yellow residue was loaded on a 5 g Sep‐Pak cartridge and the yellow product was washed down with MeOH/H_2_O (3:1, *v*/*v*). The obtained yellow ***Z*** **4**‐**Me** was dried under reduced pressure and recrystallized in CH_2_Cl_2_/*n*‐hexane. ***Z*** **4**‐**Me** was obtained as yellow microcrystals (2.3 mg, 83 % yield).

**Spectroanalytical data of pyYCC‐Me Z4‐Me**: UV/Vis (2.3×10^−4^ mol L^−1^, CHCl_3_): *λ*_max_ (log *ϵ*) 273.5 (4.20), 319 (4.41), 336 (4.28), 423.5 (4. 61), 475 (3.79); UV/Vis (1.8×10^−4^ mol L^−1^, MeOH): *λ*_max_ (log *ϵ*) 270 (4.20), 310 (4.43), 426 (4.63); CD: (2.3×10^−4^ mol L^−1^, CHCl_3_): *λ*_min/max_ (Δ*ϵ*) 290 (−4.8), 316 (−6.3), 340 (11.8), 400 (2.1), 479 nm (3.4); CD: (1.8×10^−4^ mol L^−1^, MeOH): *λ*_min/max_ (Δ*ϵ*) 282 (−2.8), 306 (1.1), 418 nm (1.1); ^1^H NMR (500 MHz, CDCl_3_, 25 °C): *δ*=1.84 (s, H_3_C2^1^), 2.12 (s, H_3_C17^1^), 2.16 (s, H_3_C13^1^), 2.30 (s, H_3_C7^1^), 2.48 (m, H_2_C12^2^), 2.68 (m, H_A_C3^1^), 2.77 (m, H_2_C12^1^), 2.81 ((dd, *J=*5.7/17.0, H_A_C8^2^), 2.88 (m, H_B_C3^1^), 3.13 (dd, *J=*6.5/17.0, H_B_C8^2^), 3.60 (m, H_A_C3^2^), 3.71 (s, H_3_C12^5^) superimposed with H_B_C3^2^, 3.80/4.07 (AB system, *J=*15.9, H_2_C5), 4.71 (t, *J=*5.7, HC10), 5.07 (d, *J=*11.6, H_A_C18^2^), 5.75 (d, *J=*17.7, H_B_C18^2^), 5.98 (dd, *J=*11.6/17.7, HC18^1^), 5.99 (s, HC15), 7.30 (s, HC20), 10.92 (s, HN23), 11.18 (s, HN24), 11.50 (s, HN22), 12.25 ppm (s, HN21); ^13^C‐NMR (CDCl_3_, 25 °C): *δ*=9.1 (2^1^), 9.9 (13^1^ and 17^1^), 10.0 (7^1^), 19.7 (12^1^), 22.1 (5), 27.3 (3^1^), 32.6 (10), 35.2 (12^2^), 50.6 (8^2^), 51.7 (12^5^), 62.7 (3^2^), 102.0 (15), 110.5 (7), 116.1 (18^2^), 118.0 (3), 121.6 (12), 122.7 (18), 123.7 (14), 126.0 (18^1^), 126.5 (13), 127.4 (8 and 16), 128.2 (1), 132.5 (6), 133.1 (11), 135.4 (2), 139.5 (4), 141.5 (17), 156.2 (9), 173.2 (12^3^), 176.4 (20), 195.1 ppm (8^1^); ^1^H NMR (500 MHz, [D_6_]DMSO, 25 °C): *δ*=2.04 (s, H_3_C13^1^), 2.06 (s, H_3_C7^1^), 2.15 (s, H_3_C17^1^), 2.17 (s, H_3_C2^1^), 2.18 (m, H_A_C12^2^), 2.29 (m, H_B_C12^2^), 2.46 (m, H_A_C12^1^), 2.47 (m, H_2_C3^1^), 2.52 (m, H_B_C12^1^), 2.67 (dd, *J=*2.8/17.3, H_A_C8^2^), 3.10 (dd, *J=*6.9/17.3, H_B_C8^2^), 3.53 (s, H_3_C12^5^), 3.29 (m, H_2_C3^2^), 3.74/3.82 (AB system, *J=*15.9, H_2_C5), 4.61 (t, *J=*4.7, HO3^3^), 4.71 (dd, *J=*2.8/6.9, HC10), 5.30 (dd, *J=*2.1/11.5, H_A_C18^2^), 6.19 (dd, *J=*2.1/17.6, H_B_C18^2^), 6.06 (s, HC15), 6.56 (dd, *J=*11.5/17.6, HC18^1^), 9.43 (s, HC20), 10.08 (s, HN24), 10.27 (s, HN23), 11.00 (s, HN22), 11.19 ppm (s, HN21); ^13^C NMR ([D_6_]DMSO, 25 °C): *δ*=8.5 (2^1^), 8.9 (7^1^ and 13^1^), 9.0 (17^1^), 18.8 (12^1^), 22.0 (5), 26.9 (3^1^), 30.8 (10), 34.2 (12^2^), 49.7 (8^2^), 51.0 (12^5^), 61.0 (3^2^), 99.4 (15), 109.1 (7), 117.2 (18^2^), 119.0 (3), 119.7 (12), 122.9 (18), 123.6 (13 and 14), 126.3 (8), 126.7 (18^1^), 127.9 (1), 128.8 (16), 131.1 (2), 132.2 (6), 133.1 (11), 136.1 (4), 142.2 (17), 157.4 (9), 170.6 (19), 172.7 (12^3^), 194.4 ppm (8^1^); ESI‐MS *m*/*z* (%): calcd for [*M*+H]^+^ C_34_H_39_N_4_O_6_: 599.3; found: 1237.2 (22), 1236.1 (30), 1235.1 (34, [2*m*+K]^+^), 1221.3 (32), 1220.3 (80), 1219.3 (100, [2*m*+Na]^+^), 1197.3 (11, [2*m*+H]^+^), 622.3 (6), 621.3 (16, [*M*+Na]^+^), 599.3 (8, [*M*+H]^+^.

### Synthesis of *E*4‐Me by *Z*/*E* photoisomerization of *Z*4‐Me

Compound ***Z*** **4**‐**Me** (2.0 mg, 3.3 μmol) was dissolved in Ar‐purged of MeOH (10 mL). The solution was illuminated by a fluorescent lamp under Ar at 0 °C. After 2 h, the isomerization of ***Z*** **4**‐**Me** to ***E*** **4**‐**Me** had reached the apparent equilibrium (1:1, detected at 320 nm by HPLC analysis). The reaction mixture was diluted with H_2_O (10 mL) and loaded on a Sep‐Pak cartridge for separation. The first yellow fraction was washed down by MeOH/H_2_O (60/40, *v*/*v*) as ***E*** **4**‐**Me**. The second yellow fraction of ***Z*** **4**‐**Me** was eluted with MeOH/H_2_O (75/25, *v*/*v*). The eluates containing ***E*** **4**‐**Me** or ***Z*** **4**‐**Me** were collected separately and concentrated under reduced pressure (to remove MeOH). Upon lyophilization, ***Z*** **4**‐**Me** (ca. 1.0 mg, 50 %) was recovered as a yellow powder, and ***E*** **4**‐**Me** (0.8 mg, 40 % yield) was obtained as a light yellow powder.

**Spectroanalytical data of** ***E*** **4‐Me**: UV/Vis (5.6×10^−4^ mol L^−1^, CHCl_3_): *λ*_max_ (log *ϵ*) 275 (4.24), 313 (4.33), 423.5 (4.26); UV/Vis (4.8×10^−4^ mol L^−1^, MeOH): *λ*_max_ (log *ϵ*) 274 (4.22), 310 (4.36), 433 (4.27); CD: (5.6×10^−4^ mol L^−1^, CHCl_3_) *λ*_min/max_ (Δ*ϵ*) 283sh (−11.5), 306 (−14.5), 340 (11.2), 385sh (0.7), 426 (−2.1), 475 nm (4.5); CD: (4.8×10^−4^ mol L^−1^, MeOH): *λ*_min/max_ (Δ*ϵ*): 280 (−2.7), 308 (1.5), 433 nm (1.3); ^1^H NMR (500 MHz, CDCl_3_, 25 °C): *δ*=1.75 (s, H_3_C17^1^), 2.06 (s, H_3_C7^1^), 2.09 (s, H_3_C13^1^), 2.15 (s, H_3_C2^1^), 2.53 (d, *J=*17.7 obtained from ^1^H,^13^C‐HSQC, H_A_C8^2^), 2.54 (m, H_A_C3^1^), 2.58 (m, H_2_C12^2^), 2.59 (m, H_B_C3^1^), 2.88 (m, H_2_C12^1^), 3.47 (dd, *J=*7.0/17.7, H_B_C8^2^), 3.66 (s, H_3_C12^5^), 3.69 (m, H_2_C3^2^), 3.84/3.95 (AB system, *J=*18.5, H_2_C5), 4.74 (d, *J=*7.0, HC10), 5.20 (d, *J=*11.7, H_A_C18^2^), 5.73 (s, HN24), 5.89 (d, *J=*18.3, H_B_C18^2^), 5.91 (s, HC15), 6.07 (dd, *J=*11.7/18.3, HC18^1^), 8.76 (s, HC20), 9.24 (s, HN21), 9.50 (s, HN23), 10.66 ppm (s, HN22); ^13^C NMR (CDCl_3_, 25 °C): *δ*=8.9 (2^1^), 9.1 (7^1^), 10.0 (13^1^), 12.1 (17^1^), 19.6 (12^1^), 23.1 (5), 27.0 (3^1^), 31.4 (10), 35.4 (12^2^), 51.8 (12^5^), 52.5 (8^2^), 61.9 (3^2^), 107.9 (15), 112.5 (7), 119.2 (18^2^), 119.9 (3), 120.5 (12 and 14), 123.0 (13), 125.2 (18^1^), 127.9 (8), 128.2 (1 and 18), 130.3 (6), 133.3 (11), 134.5 (16), 135.9 (2), 137.6 (17), 140.9 (4), 156.9 (9), 170.0 (19), 173.4 (12^3^), 175.4 (20), 195.7 ppm (8^1^); ESI‐MS *m*/*z* (%): calcd for [*M*+H]^+^ C_34_H_39_N_4_O_6_: 599.3; found: 1237.0 (25), 1236.1 (46), 1235.2 (58, [2 *m*+K]^+^), 1222.2 (20), 1221.2 (41), 1220.2 (96), 1219.2 (100, [2*M*+Na]^+^), 1198.1 (14), 1197.1 (15, [2*m*+H]^+^), 637.3 (11, [*M*+K]^+^), 622.2 (6), 621.2 (20, [*M*+Na]^+^), 599.1 (10, [*M*+H]^+^).

### Synthesis of 5**‐**Me by photodimerization of *Z*4**‐**Me

Crystalline ***Z*** **4**‐**Me** (2.6 mg, 4.3 μmol) was dissolved in 0.4 mL of CDCl_3_ in an NMR tube. The solution was purged with Ar for 5 min and irradiated with the fluorescence lamp under Ar at 0 °C. After 8 h, the conversion of ***Z*** **4**‐**Me** to **5**‐**Me** was complete, based on NMR analysis (purity >95 %). The solvent was removed by a stream of Ar, furnishing 2.6 mg of **5**‐**Me** as a pale yellow residue.

**Spectroanalytical data**: UV/Vis (1.4×10^−4^ mol L^−1^, CHCl_3_): *λ*_max_ (log *ϵ*) 323 (4.59), 268 (4.56); CD (1.4×10^−4^ mol L^−1^, CHCl_3_): *λ*_min/max_ (Δ*ϵ*) 277 (−17.8), 317 (−13.5), 340 nm (22.3); ^1^H NMR (500 MHz, CDCl_3_, 25 °C): *δ*=1.74 (s, H_3_C13^1^), 1.99 (s, H_3_C7^1^), 2.22 (s, H_3_C17^1^), 2.30 (m, H_A_C12^2^), 2.33 (s, H_3_C2^1^), 2.46 (m, H_B_C12^2^), 2.58 (m, H_A_C3^1^), 2.66 (dd, *J=*3.6/18.0, H_A_C8^2^), 2.67 (m, H_B_C3^1^), 2.71 (m, H_2_C12^1^), 3.20 (dd, *J=*6.8/18.0, H_B_C8^2^), 3.44 (s, H_3_C12^5^), 3.67 (m, H_A_C3^2^), 3.72 (m, H_B_C3^2^), 3.73/4.14 (AB system, *J=*18.8, H_2_C5), 4.33 (s, HC15), 4.49 (dd, *J=*3.6/6.8, HC10), 5.35 (d, *J=*11.6, H_A_C18^2^), 6.05 (d, *J=*17.7, H_B_C18^2^), 6.25 (dd, *J=*11.6/17.7, HC18^1^), 6.78 (s, HN24), 8.72 (s, HC20), 9.87 (s, HN21), 10.55 (s, HN23), 10.63 ppm (s, HN22); ^13^C NMR (CDCl_3_, 25 °C): *δ*=8.9 (7^1^), 9.1 (2^1^ and 13^1^), 10.9 (17^1^), 19.5 (12^1^), 22.9 (5), 28.9 (3^1^), 31.2 (10), 36.2 (12^2^), 43.5 (15), 51.0 (8^2^), 51.7 (12^5^), 61.8 (3^2^), 73.2 (16), 112.6 (7), 117.4 (12 and 13), 118.2 (14), 120.7 (3), 120.8 (18^2^), 124.9 (18^1^), 127.2 (8), 127.4 (11), 128.4 (18), 128.6 (1), 130.1 (6), 136.4 (2), 142.2 (4), 150.6 (17), 159.2 (9), 172.6 (19), 173.9 (12^3^), 176.6 (20), 195.9 ppm (8^1^); ESI‐MS *m*/*z* (%): calcd for [*M*+H]^+^ C_68_H_77_N_8_O_12_: 1197.6; found: 1236.4 (41), 1235.5 (35, [*M*+K]^+^), 1121.3 (36), 1120.3 (78), 1219.4 (100, [*M*+Na]^+^), 1197.4 (10, [*M*+H]^+^).

### Kinetic analysis of the thermolysis of 5**‐**Me to *Z*4**‐**Me

A stock solution (1.78×10^−4^ mol L^−1^) of ***Z*** **4**‐**Me** in acid‐free CHCl_3_ was prepared. A 1 mm UV/Vis cell was filled with 0.2 mL of the stock solution and purged with Ar for 1 min. The solution was then irradiated at 0 °C by the fluorescent lamp for 120 min, until light absorption at 420 nm was minimal, indicating full conversion to **5**‐**Me**. Subsequently, the solution of **5**‐**Me** was left at 23 °C in the darkness for certain time. Light absorption at 420 nm was employed to monitor the decomposition reaction. Two further experiments were performed in parallel at 40 °C and at 50 °C, in order to observe the temperature dependence of the decomposition reaction of **5**‐**Me**. The decomposition kinetics were monitored on the basis of the absorption at 420 nm.

### Computational details

The experimental X‐ray crystal structure of pyYCC‐Me (***Z*** **4**‐**Me**)_**2**_ was used as a starting structure for the quantum chemical investigation. The structure was fully optimized with density functional theory when employing the BP86^44^ density functional in combination with the triple‐zeta basis set def2‐TZVP^45^ and the resolution‐of‐identity technique.^46^ Incorporation of empirical dispersion corrections of the D3 type by Grimme^47^ were required to reproduce the experimental (***Z*** **4**‐**Me**)_**2**_ structure and obtain the correct distance for the π–π stacking of rings C and D. D3 corrections were used for all structure optimizations. All calculations were performed with the program suite Turbomole.^48^

The starting structure of pyro‐photodimer **5‐Me** was created by manual modification of (***Z*** **4‐Me**)_**2**_ in MOE.^49^ As no crystal data was available, various conformations were generated by Maestro′s conformational search tool^50^ und subsequently optimized with BP86/RI/def2‐TZVP/D3. The same procedure was applied to pyYCC‐Me monomer ***Z*** **4**‐**Me** and ***E*** **4**‐**Me**, which were not observed as monomers in apolar solvents.

To ensure all reported structures represent energy minima on the potential energy surface, vibrational spectra were calculated with Turbomole's NumForce module at the BP86/RI/def2‐TZVP/D3 level.

Solvent effects are neglected because only implicit solvent corrections are feasible and they are expected to be small in apolar solvents. Zero‐point energies and thermal corrections to electronic energies were obtained by Turbomole's freeh tool. Reported reaction energies are Gibb's free energies at standard conditions.

Recently published similar structures of the natural yellow phyllobilin dimer YCC‐Me named (***Z*** **1**‐**Me**)_**2**_ 31 and the corresponding photodimer, named **2**‐**Me** here, as well as the monomer units named ***Z*** **1**‐**Me** and ***E*** **1**‐**Me**, respectively,^31^ have been generated and optimized with the same quantum chemical protocol (for details see Ref. 31).

Structures were visualized using PyMOL.^51^ Overlay structures of pyYCC‐Me dimer **(*Z*4**‐**Me)_2_** and YCC‐Me dimer (***Z*** **1**‐**Me**)_**2**_ were created by alignment of rings C and D (see the Supporting Information, Figure S18). To determine the angle between rings B and E, E and CD as well as B and CD, Amber's cpptraj tool^52^ was used.

### X‐ray crystal structure analysis

**Single crystals of Z4‐Me**: A solution of the crystalline ***Z*** **4**‐**Me** (5 mg) was dissolved in CHCl_3_ (2.5 mL). The solution was filtered through a tight plug of cotton wool and the filtrate was collected in a 5 mL vessel. Hexane (2.5 mL) was slowly added above CHCl_3_ to form two layers. After ca. 1 week, single crystals were obtained.

For collection of X‐ray data of a single crystal of ***Z*** **4**‐**Me** with Mo radiation, a Bruker D8 Quest diffractometer was used controlled by the APEX2 software. Data integration and reduction were performed using the SAINT software.^53^ The crystal structure was solved and refined with SHELXT^54^ and SHELXL,^55^ respectively. Further details are described in the Supporting Information.

CCDC 1581089 contains the supplementary crystallographic data for this paper. These data are provided free of charge by The Cambridge Crystallographic Data Centre.

## Conflict of interest

The authors declare no conflict of interest.

## Supporting information

As a service to our authors and readers, this journal provides supporting information supplied by the authors. Such materials are peer reviewed and may be re‐organized for online delivery, but are not copy‐edited or typeset. Technical support issues arising from supporting information (other than missing files) should be addressed to the authors.

Supplementary
