# Supplementary material for: Pyro‐Phyllobilins: Elusive Chlorophyll Catabolites Lacking a Critical Carboxylate Function of the Natural Chlorophylls
Source: Chemistry. 2018 Jan 31;24(12):2987–98. doi: 10.1002/chem.201705331 (PMC5861670; doi:10.1002/chem.201705331)
Supplement: Supplementary file 1 — Supplementary [file CHEM-24-2987-s001.pdf]

CHEMISTRY
A European Journal

Supporting Information

Pyro-Phyllobilins: Elusive Chlorophyll Catabolites Lacking a Critical Carboxylate Function of the Natural Chlorophylls

Chengjie Li,[a, c] Klaus Wurst,[b] Joachim Berghold,[a] Maren Podewitz,[b] Klaus R. Liedl,[b] and Bernhard Kräutler*[a]

chem_201705331_sm_miscellaneous_information.pdf

Table S1. 500 MHz ^{1}H NMR data of pyYCC **4** and of YCC [a)] in CD_3OD. Significant chemical shift differences are highlighted by bold numbers.

	**4**		**YCC**
H_3C-2^1	2.21	H_3C-2^1	2.23
H_2C-3^1	2.61	H_2C-3^1	2.63
H_2C-3^2	3.44	H_2C-3^2	3.48
H_2C-5	3.91 / 3.94 (AB system)	H_2C-5	3.96
H_3C-7^1	2.13	H_3C-7^1	2.13
H_2C-8^2	**2.89 / 3.24**	HC-8^2	**H/D-exchange with CD_3OD**
		H_3C-8^5	**3.77**
HC-10	**4.75**	HC-10	**5.05**
H_2C-12^1	2.71 / 2.80	H_2C-12^1	2.71 / 2.79
H_2C-12^2	2.35	H_2C-12^2	2.35
H_3C-13^1	2.15	H_3C-13^1	2.15
HC-15	6.21	HC-15	6.21
H_3C-17^1	2.19	H_3C-17^1	2.20
HC-18^1	6.54	HC-18^1	6.55
H_2C-18^{2trans}	6.09	H_2C-18^{2trans}	6.11
H_2C-18^{2cis}	5.32	H_2C-18^{2cis}	5.35
HC-20	9.24 (broad singlet)	HC-20	

a) Data from Moser, S.; Ulrich, M.; Müller, T.; Kräutler, B., A yellow chlorophyll catabolite is a pigment of the fall colours. *Photochem. Photobiol. Sci.* **2008**, *7*, 1577-1581.

Table S2. Crystal data and structure refinement for ***Z*4-Me**.

Empirical formula	C_{68} H_{73} N_8 O_{12} x 0.5 C_6H_{14}	
Formula weight	1240.45	
Temperature	203(2) K	
Wavelength	0.71073 Å	
Crystal system	Monoclinic	
Space group	$P2_1$ (no. 4)	
Unit cell dimensions	a = 19.731(3) Å	α= 90°
	b = 8.957(2) Å	β= 116.247(4)°
	c = 21.832(4) Å	γ = 90°
Volume	3460.6(11) $Å^3$	
Z	2	
Density (calculated)	1.190 Mg/cm^3	
Absorption coefficient	0.082 mm^{-1}	
F(000)	1322	
Crystal size	0.200 x 0.200 x 0.030 mm^3	
Theta range for data collection	2.501 to 20.498°.	
Index ranges	-19<=h<=19, -8<=k<=8, -21<=l<=21	
Reflections collected	20910	
Independent reflections	6923 [R(int) = 0.0810]	
Completeness to theta = 20.498°	99.6 %	
Absorption correction	Semi-empirical from equivalents	
Max. and min. transmission	0.992 and 0.865	
Refinement method	Full-matrix least-squares on F^2	
Data / restraints / parameters	6923 / 19 / 845	
Goodness-of-fit on F^2	1.044	
Final R indices [I>2sigma(I)]	R1 = 0.0689, wR2 = 0.1665	
R indices (all data)	R1 = 0.0927, wR2 = 0.1786	
Absolute structure parameter	-0.7(10)	
Largest diff. peak and hole	0.267 and -0.281 e.$Å^{-3}$	

Table S3. Hydrogen bonds observed in the crystal structure of ***Z*4-Me**.

D-H...A	d(D-H) / Å	d(H...A) / Å	d(D...A) / Å	<(DHA) / ^{o}
N(21)-H(21)...O(591)	0.87	2.02	2.867(10)	163.7
N(22)-H(22)...O(591)	0.87	1.95	2.768(10)	155.4
N(23)-H(23)...O(601)	0.87	1.94	2.808(10)	173.9
N(24)-H(24)...O(601)	0.87	1.89	2.756(11)	173.2
N(61)-H(61)...O(191)	0.87	2.01	2.852(10)	162.3
N(62)-H(62)...O(191)	0.87	1.95	2.758(11)	153.1
N(63)-H(63)...O(201)	0.87	1.98	2.845(11)	171.7
N(64)-H(64)...O(201)	0.87	1.90	2.763(11)	173.0
O(33)-H(33)...O(811)#1	0.83	1.98	2.797(11)	169.9
O(433)-H(433)...O(481)#2	0.83	1.98	2.806(11)	174.7

Symmetry transformations used to generate equivalent atoms: #1 x,y+1,z; #2 x,y-1,z

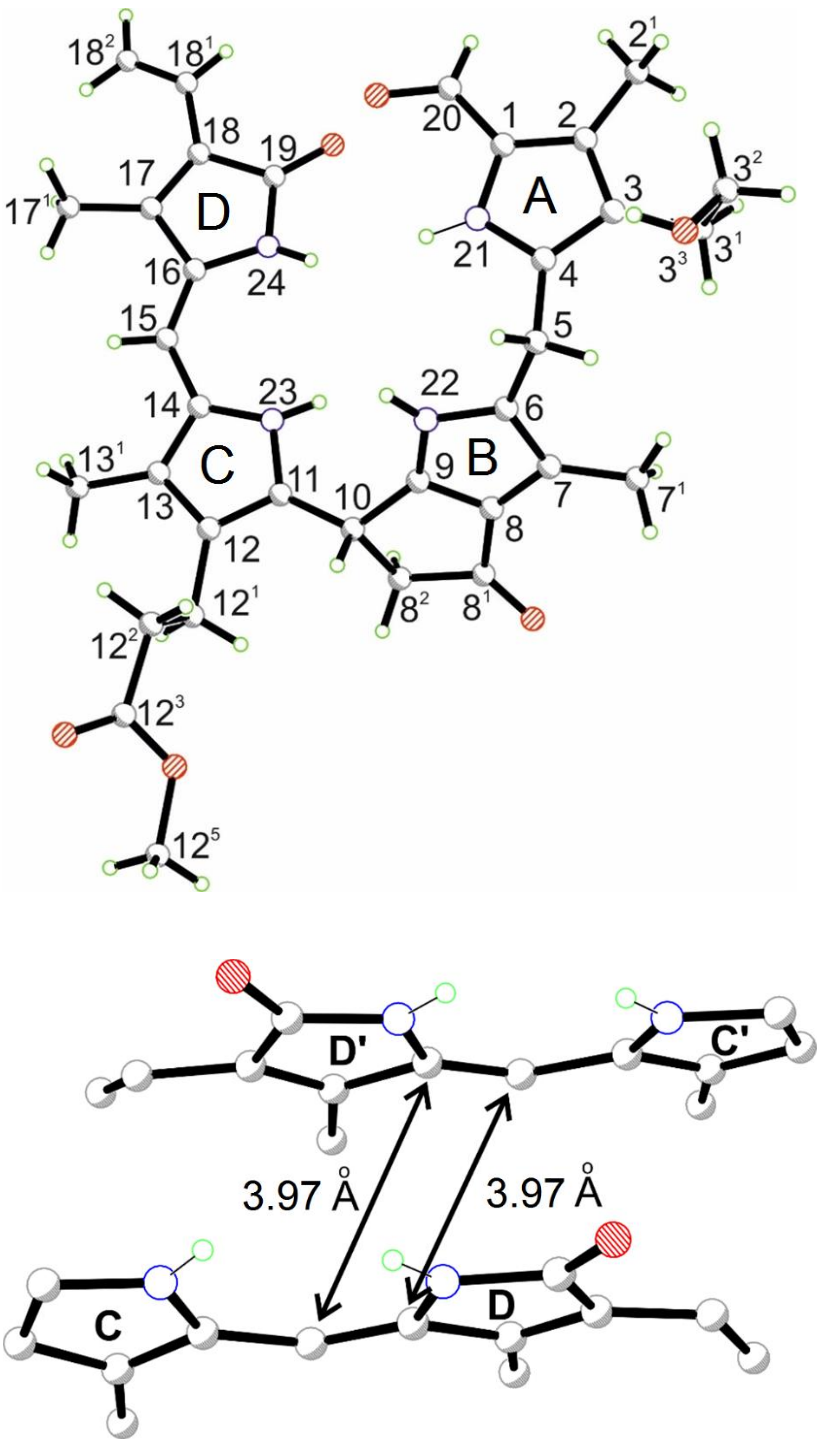

Figure S1. Top: Ball and stick model of the crystal structure of ***Z*4-Me** with atom numbering (top). Bottom: Structures of the C/D-moieties of ***Z*4-Me** in the non-covalent dimer structure (***Z*4-Me**)$_2$ and distances between the carbons atoms (C15-C16’ and C15’-C16), between which the new bonds are formed in the [2+2]-cycloaddition reaction (other parts of (***Z*4-Me**)$_2$ were deleted for clarity)

Table S4. ^{1}H- and ^{13}C-NMR signal assignments of ***Z*4-Me**, ***E*4-Me** and **5-Me** from 500 MHz NMR spectra in $CDCl_3$.

	***Z*4-Me** ^{1}H [a]	***Z*4-Me** ^{13}C	***E*4-Me** ^{1}H [a]	***E*4-Me** ^{13}C	**5-Me** ^{1}H [a]	**5-Me** ^{13}C
C-1		128.2		128.2		128.6
C-2		135.4		135.9		136.4
H_3C-2^1	1.84 (s)	9.1	2.15 (s)	8.9	2.33 (s)	9.1
C-3		118.0		119.9		120.7
H_2C-3^1	2.68 / 2.88 (m)	27.3	2.54 / 2.59 (m)	27.0	2.58 / 2.67 (m)	28.9
H_2C-3^2	3.60 / 3.71 (m)	62.7	3.69 (m)	61.9	3.67 / 3.72 (m)	61.8
C-4		139.5		140.9		142.2
H_2C-5	3.80 / 4.07 (AB, 15.9)	22.1	3.84 / 3.95 (AB, 18.5)	23.1	3.73 / 4.14 (AB, 18.8)	22.9
C-6		132.5		130.3		130.1
C-7		110.5		112.5		112.6
H_3C-7^1	2.30 (s)	10.0	2.06 (s)	9.1	1.99 (s)	8.9
C-8		127.4		127.9		127.2
C-8^1		195.1		195.7		195.9
H_AC-8^{2trans}	2.81 (dd, 5.7, 17.0)	50.6	2.53 (d, 17.7[b])	52.5	2.66 (dd, 3.6 / 18.0[b])	51.0
H_BC-8^{2cis}	3.13 (dd, 6.5, 17.0)	50.6	3.47 (dd, 7.0 / 17.7)	52.5	3.20 (dd, 6.8 / 18.0)	51.0
C-9		156.2		156.9		159.2
HC-10	4.71 (t, 5.7)	32.6	4.74 (d, 7.0)	31.4	4.49 (dd, 3.6 / 6.8)	31.2
C-11		133.1		133.3		127.4
C-12		121.6		120.5		117.4
H_2C-12^1	2.77 (m)	19.7	2.88 (m)	19.6	2.71 (m)	19.5
H_2C-12^2	2.48 (m)	35.2	2.58 (m)	35.4	2.30 / 2.46 (m)	36.2
C-12^3		173.2		173.4		173.9
H_3C-12^5	3.71 (s)	51.7	3.66 (s)	51.8	3.44 (s)	51.7
C-13		126.5		123.0		117.4
H_3C-13^1	2.16 (s)	9.9	2.09 (s)	10.0	1.74 (s)	9.1
C-14		123.7		120.5		118.2
HC-15	5.99 (s)	102.0	5.91 (s)	107.9	4.33 (s)	43.5
C-16		127.4		134.5		73.2
C-17		141.5		137.6		150.6
H_3C-17^1	2.12 (s)	9.9	1.75 (s)	12.1	2.22 (s)	10.9
C-18		122.7		128.2		128.4
HC-18^1	5.98 (dd, 11.6 / 17.7)	126.0	6.07 (dd, 11.7 / 18.3)	125.2	6.25 (dd, 11.6 / 17.7)	124.9
H_2C-18^{2trans}	5.75 (d, 17.7)	116.1	5.89 (d, 18.3)	119.2	6.05 (d, 17.7)	120.8
H_2C-18^{2cis}	5.07 (d, 11.6)	116.1	5.20 (d, 11.7)	119.2	5.35 (d, 11.6)	120.8
C-19				170.0		172.6
HC-20	7.30 (s)	176.4	8.76 (s)	175.4	8.72 (s)	176.6
HN21	12.25 (s)		9.24 (s)		9.87 (s)	
HN22	11.50 (s)		10.66 (s)		10.63 (s)	
HN23	10.92 (s)		9.50 (s)		10.55 (s)	
HN24	11.18 (s)		5.73 (s)		6.78 (s)	

[a] chemical shift (signal type, coupling constant in Hz); [b] coupling constant was obtained from ^{1}H,^{13}C-HSQC spectra.

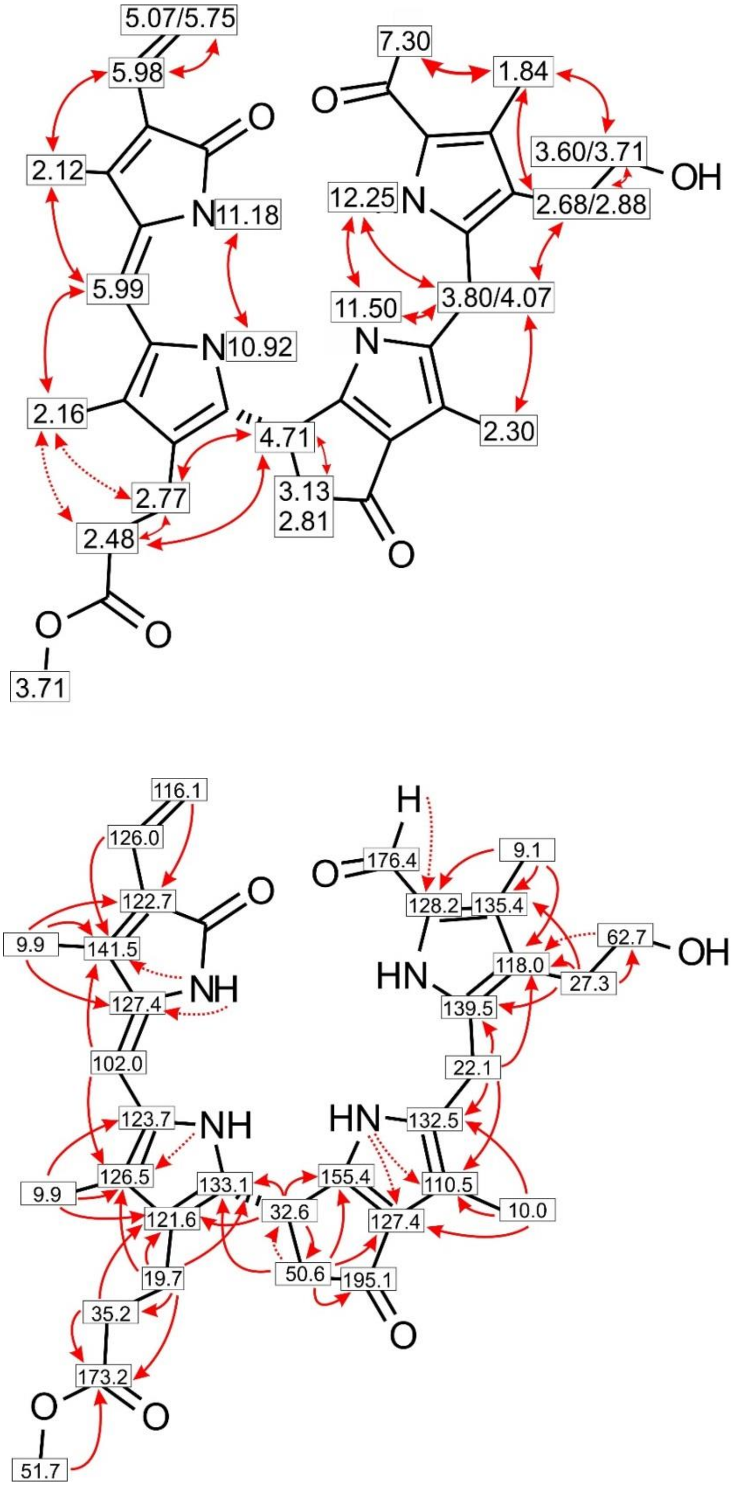

Figure S2. NMR analysis of (***Z*4-Me**)$_2$ in $CDCl_3$ (500 MHz, 25 °C). Top. Intra-modular ^{1}H,^{1}H-correlations from ROESY spectra. Bottom. ^{1}H,^{13}C-heteronuclear correlations from HSQC and HMBC spectra.

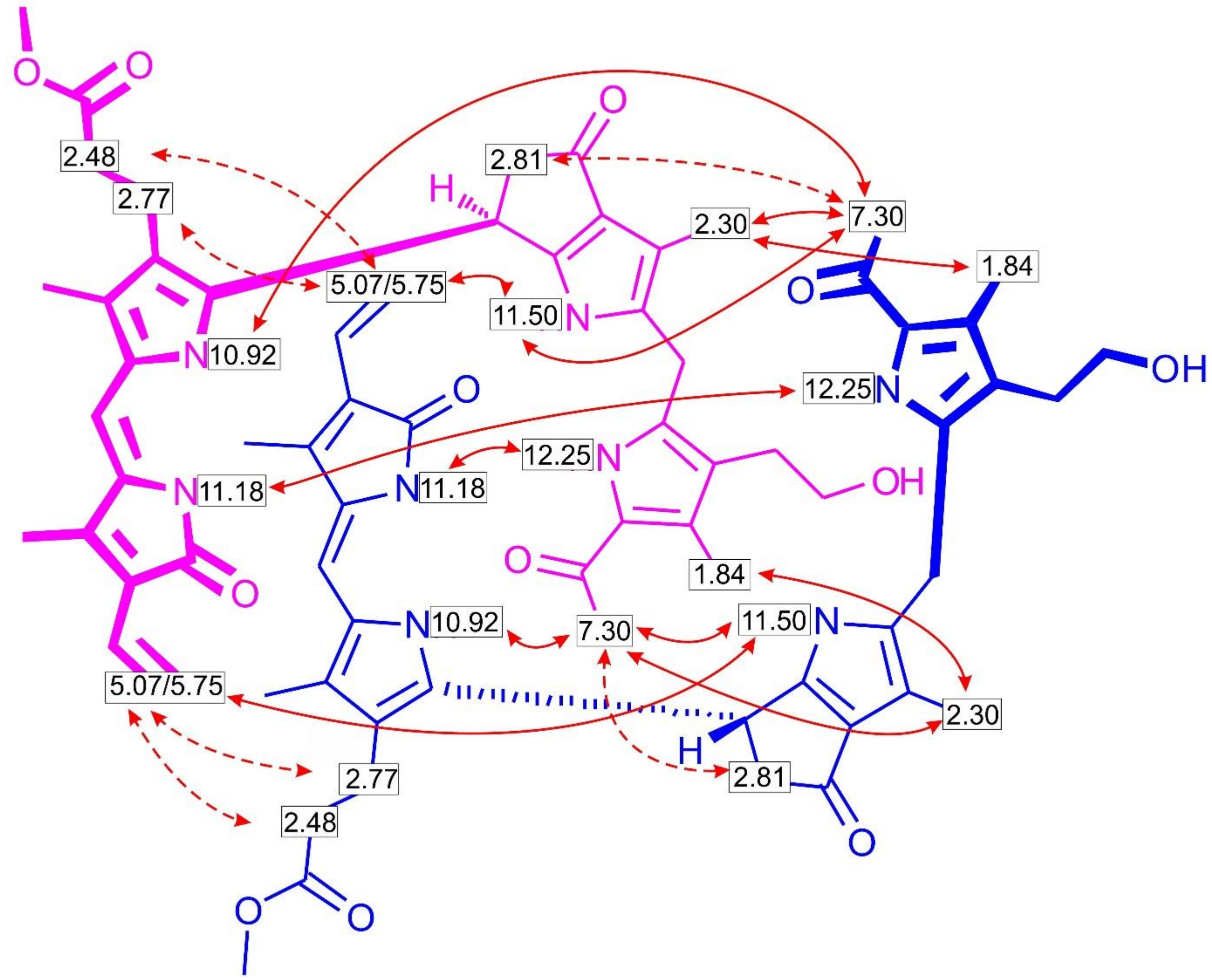

Figure S3. Inter-modular homonuclear ^{1}H,^{1}H-correlations of H-bonded homodimer (***Z*4-Me)₂** from ROESY spectra in $CDCl_3$ (500 MHz, 25 °C).

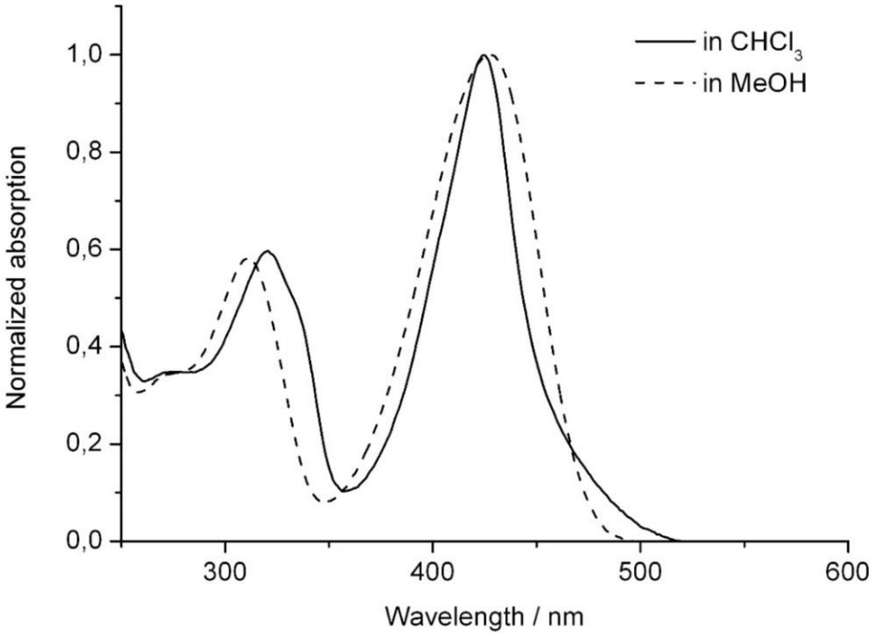

Figure S4. UV/Vis spectra of ***Z*4-Me** in $CHCl_3$ and MeOH (normalized at maxima near 425 nm) indicate ***Z*4-Me** to occur as a non-covalent H-bonded dimer or as a monomer, respectively.

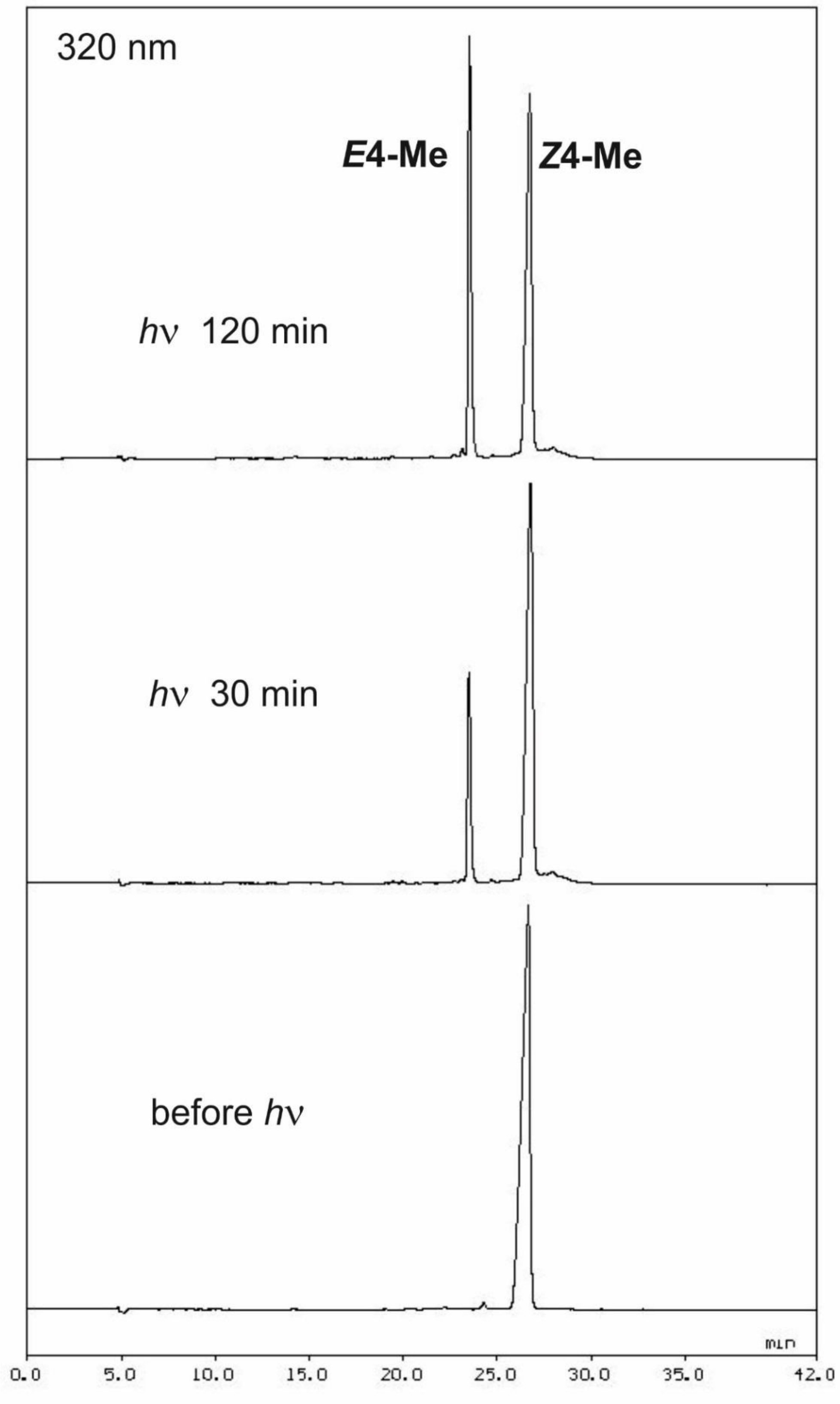

Figure S5. *Z*/*E*-photoisomerization of ***Z*4-Me**. HPLC-analysis of a solution of ***Z*4-Me** (8.4×10^{-4} M) in MeOH before and after irradiation by the fluorescence lamp at 0°C.

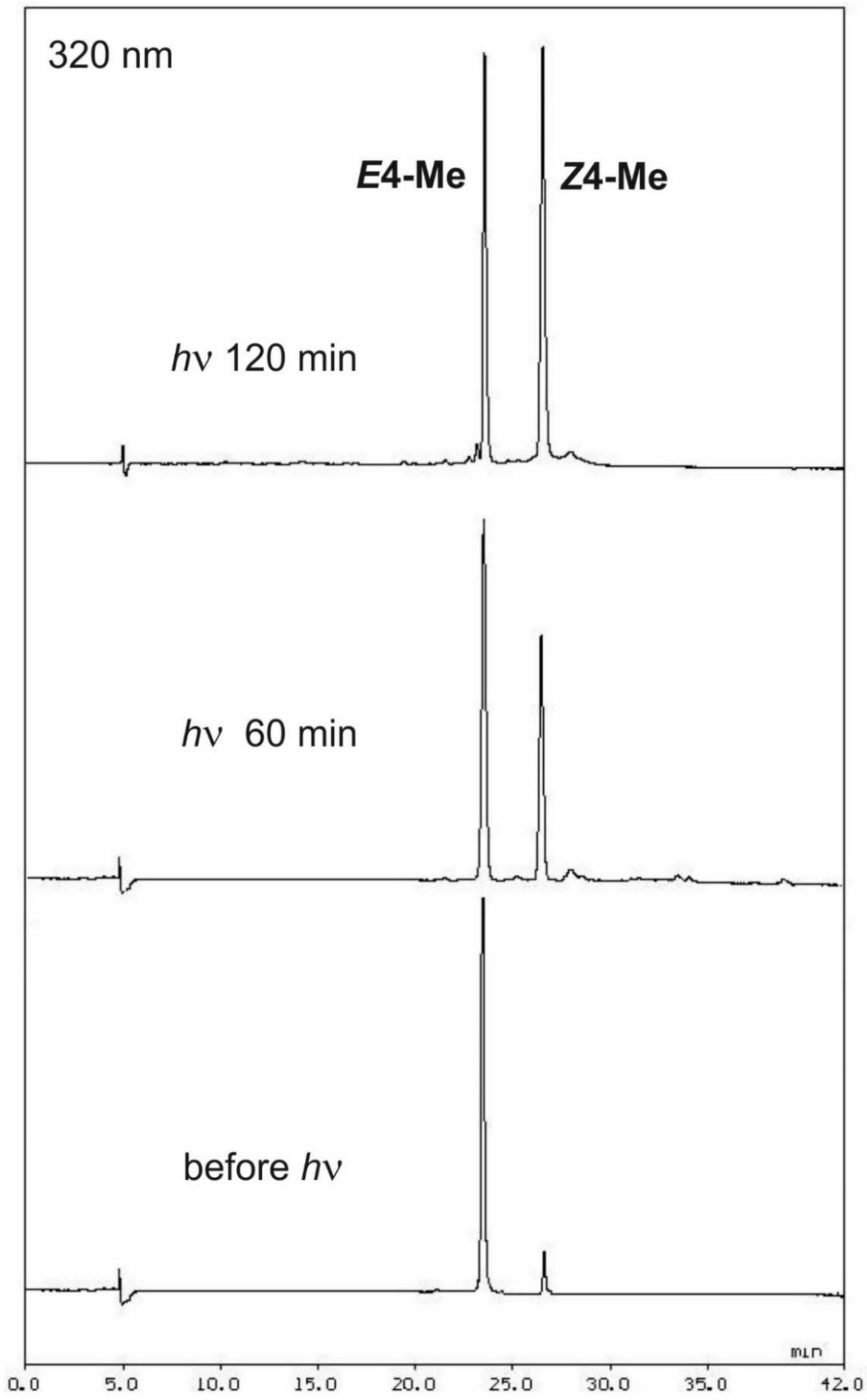

Figure S6. *E*/*Z*-photoisomerization of ***Z*4-Me**. HPLC-analysis of a solution of ***Z*4-Me** (4.4×10^{-4} M) in MeOH before and after irradiation by the fluorescence lamp at 0°C

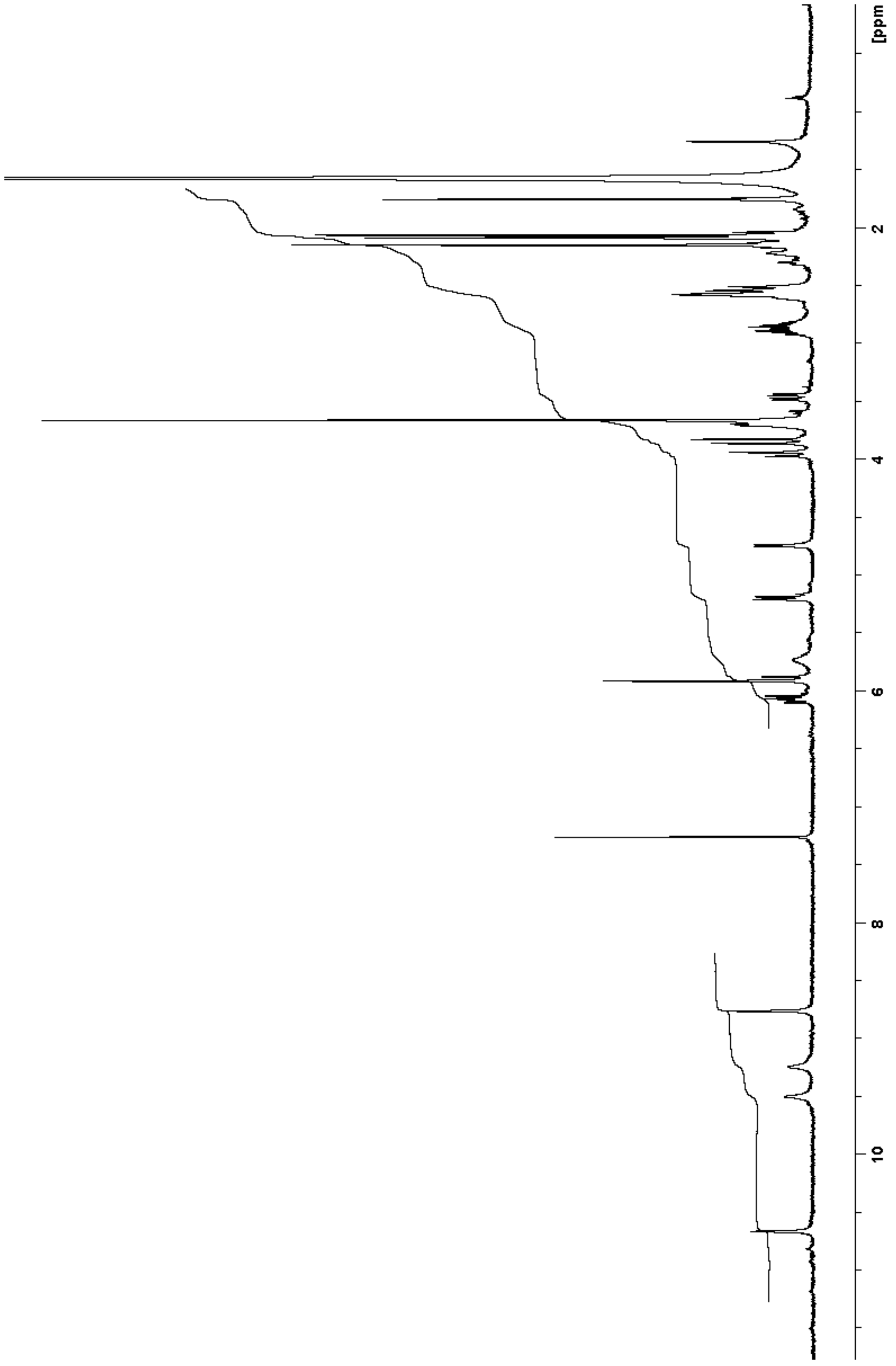

Figure S7. 500 MHz ^{1}H NMR spectrum of ***E*4-Me** in $CDCl_3$ (2.7×10^{-3} M, 25 °C)

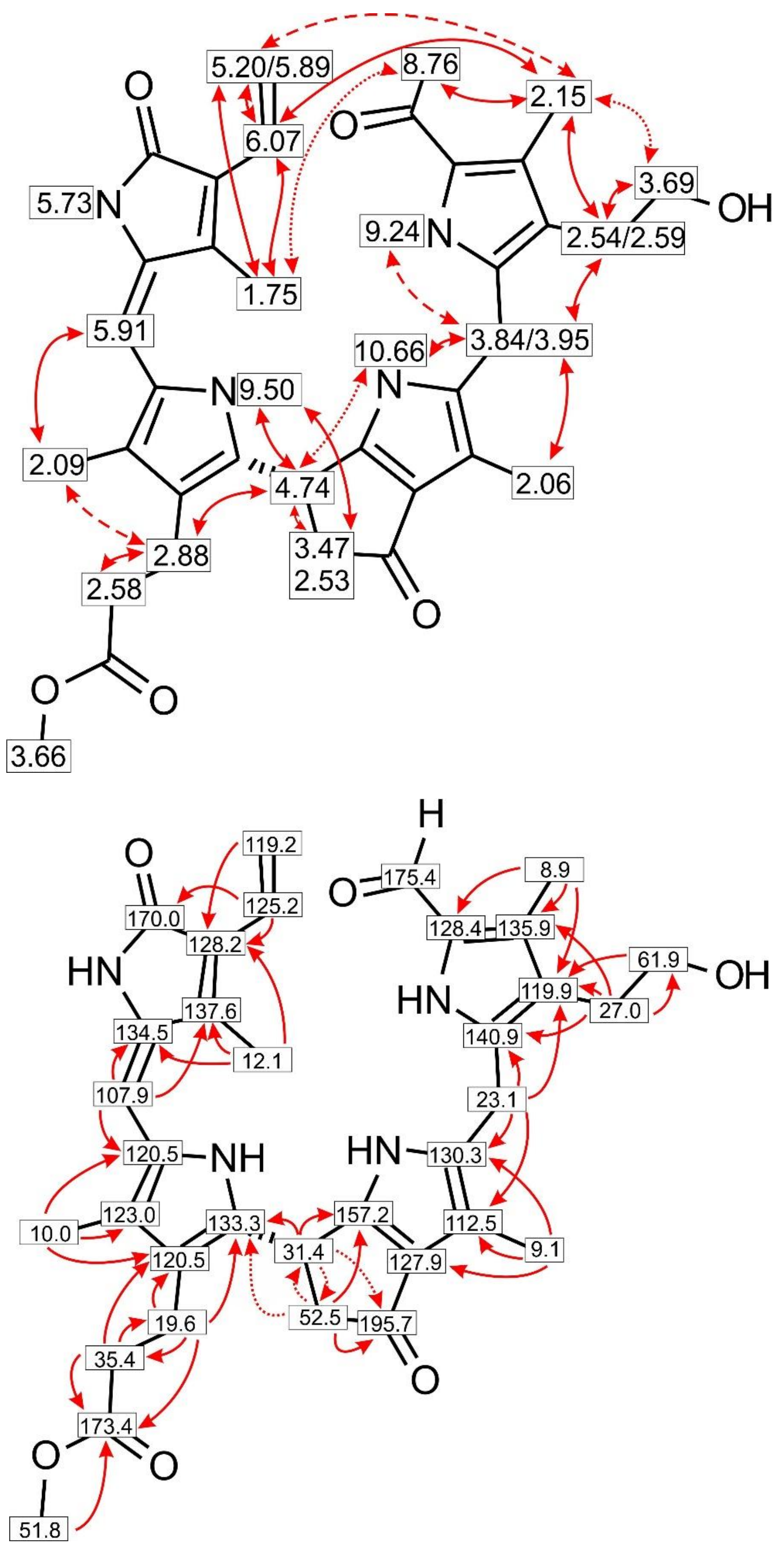

Figure S8. Structural analysis of ***E*4-Me** by NMR (500 MHz, $CDCl_3$, 25°C). Top. $^1H,^1H$ homonuclear correlations from a ROESY spectrum. Bottom. $^1H,^{13}C$ heteronuclear correlations from HSQC- and HMBC-spectra.

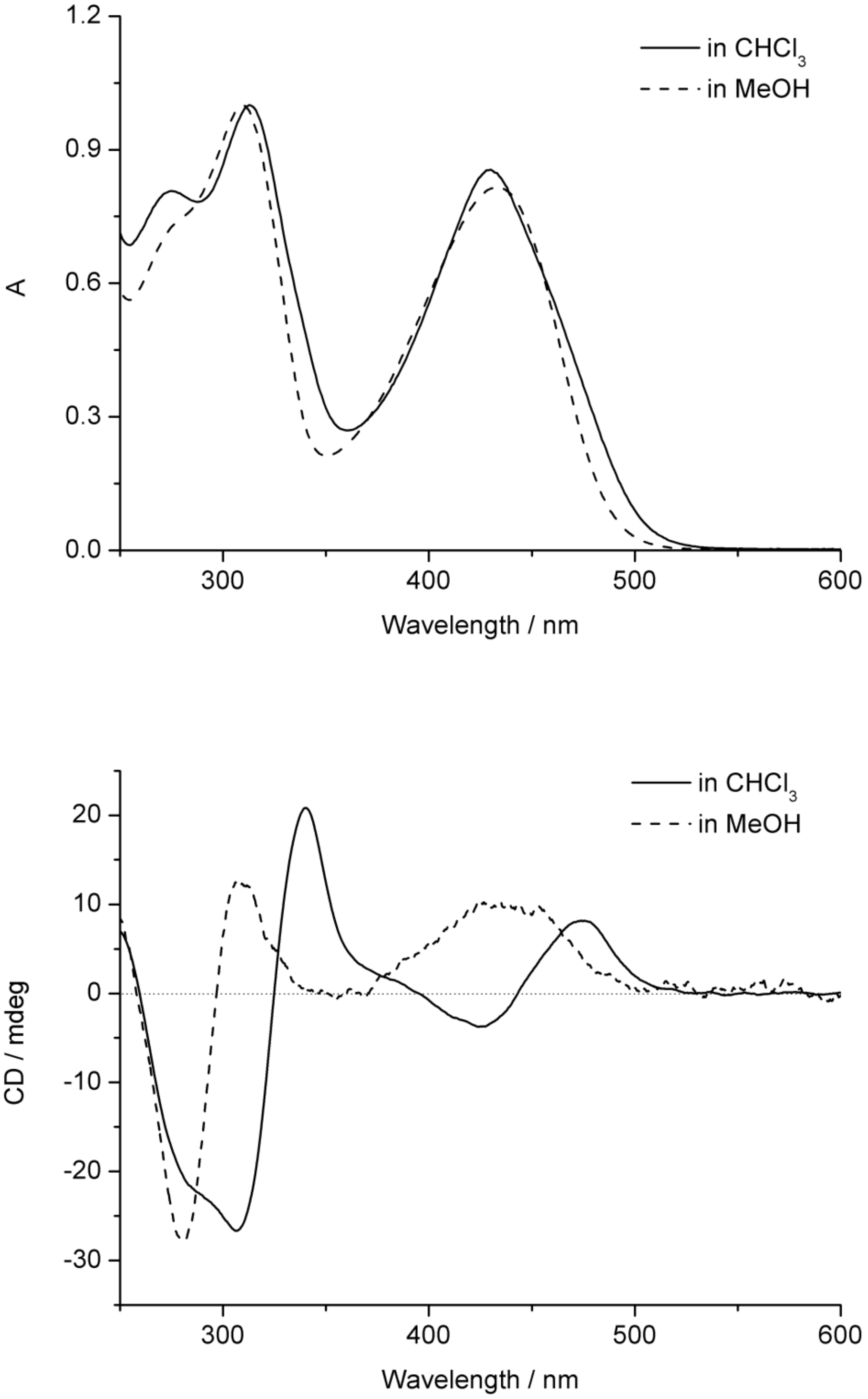

Figure S9. Top. UV/Vis spectra of ***E*4-Me** in $CHCl_3$ (5.6×10^{-4} M, solid line) and in MeOH (4.8×10^{-4} M, dashed line), both normalized at their maximum near 320 nm, respectively. Bottom. CD spectra of ***E*4-Me** as a 5.6×10^{-4} M solution in $CHCl_3$ (solid line) and as a 4.8×10^{-3} M solution in MeOH (dashed line). The y-axis for the CD spectra of the two solutions of ***E*4-Me** was adjusted for a better comparison.

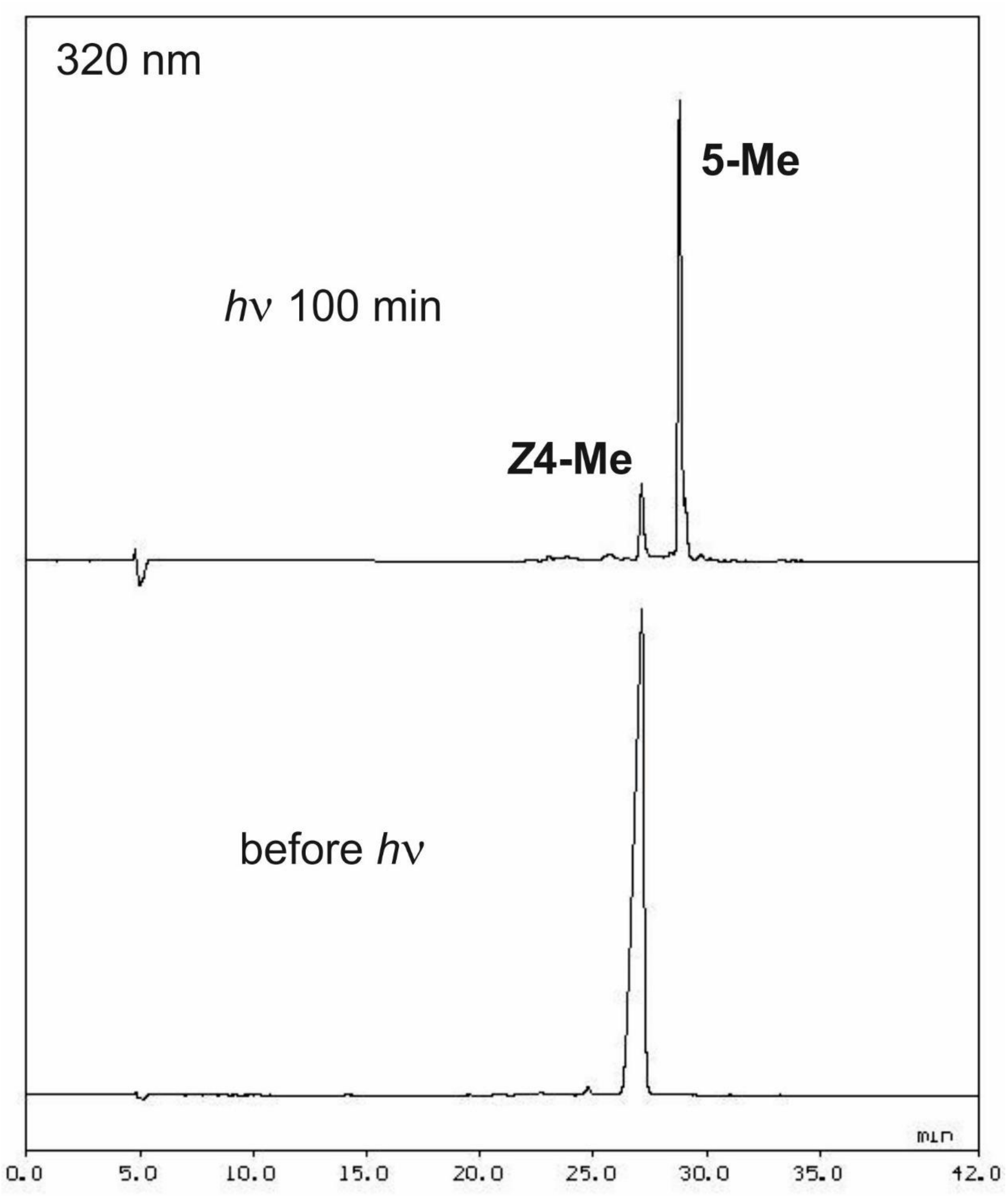

Figure S10. Photodimerization of ***Z*4-Me** in $CDCl_3$. HPLC-analysis of the solution of ***Z*4-Me** (1.8×10^{-4} M) before, and after irradiation by the fluorescence lamp at 0°C.

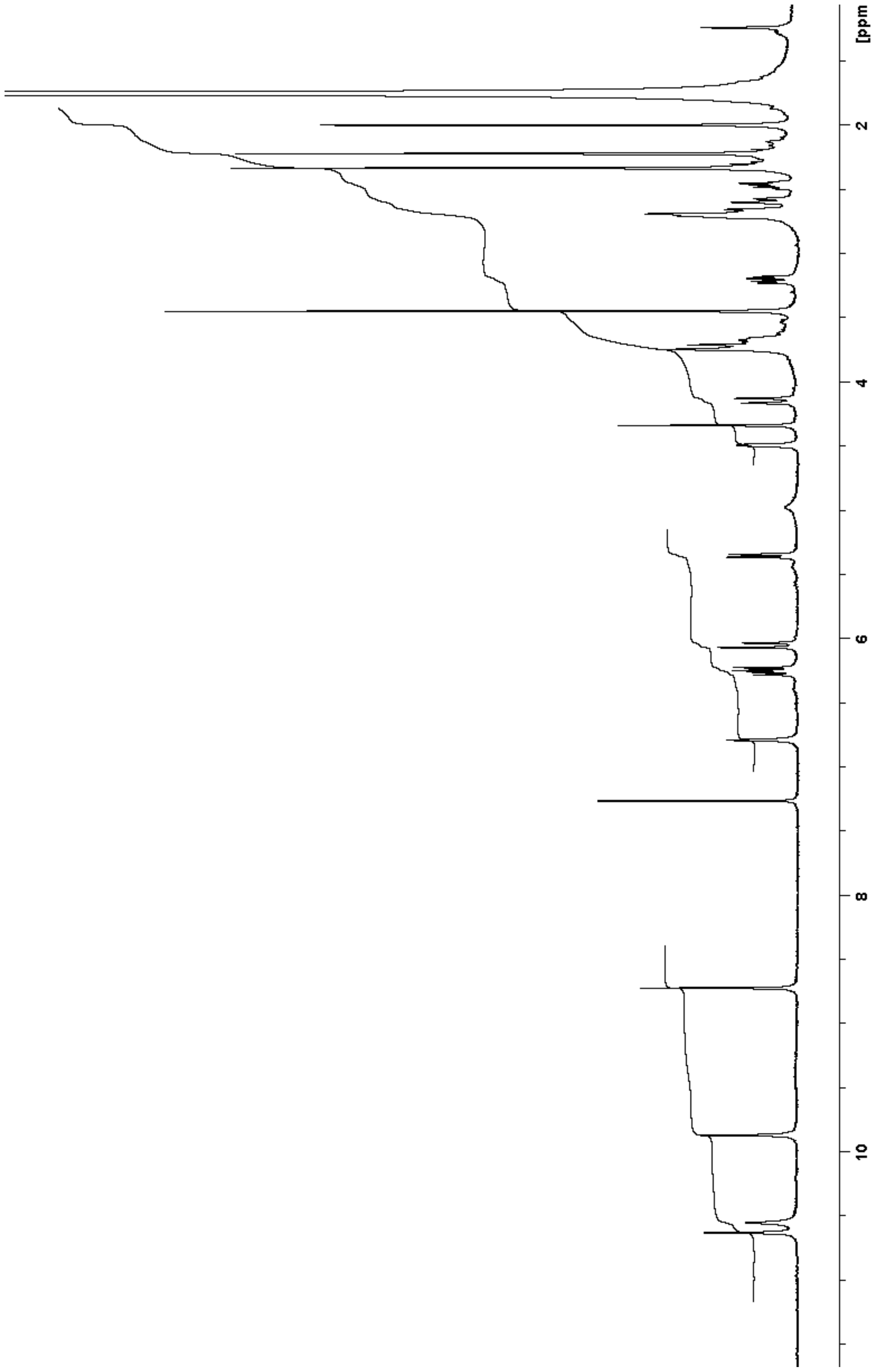

Figure S11. ^{1}H NMR spectrum of **5-Me** in $CDCl_3$ (1.3×10^{-3} M, 500 MHz, 0 °C)

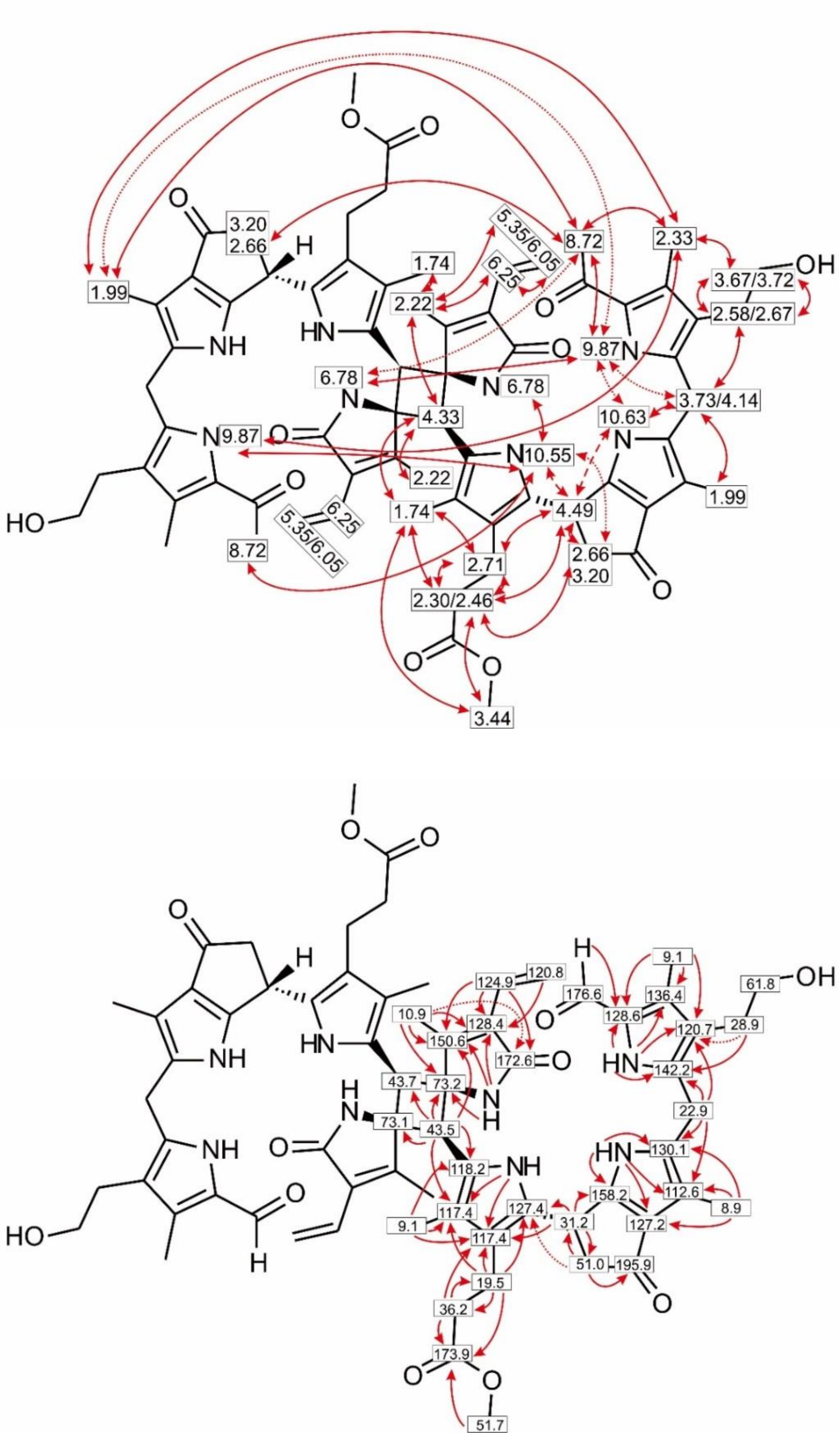

Figure S12. Structure analysis of covalent dimer **5-Me** by $^{1}H,^{1}H$-homonuclear and $^{1}H,^{13}C$-heteronuclear NMR spectra in $CDCl_3$ (at 500 MHz, 0 °C). Top. Set of homonuclear correlations from a $^{1}H,^{1}H$-ROESY spectrum. Bottom. Set of heteronuclear correlations from $^{1}H,^{13}C$-HSQC and $^{1}H,^{13}C$-HMBC spectra.

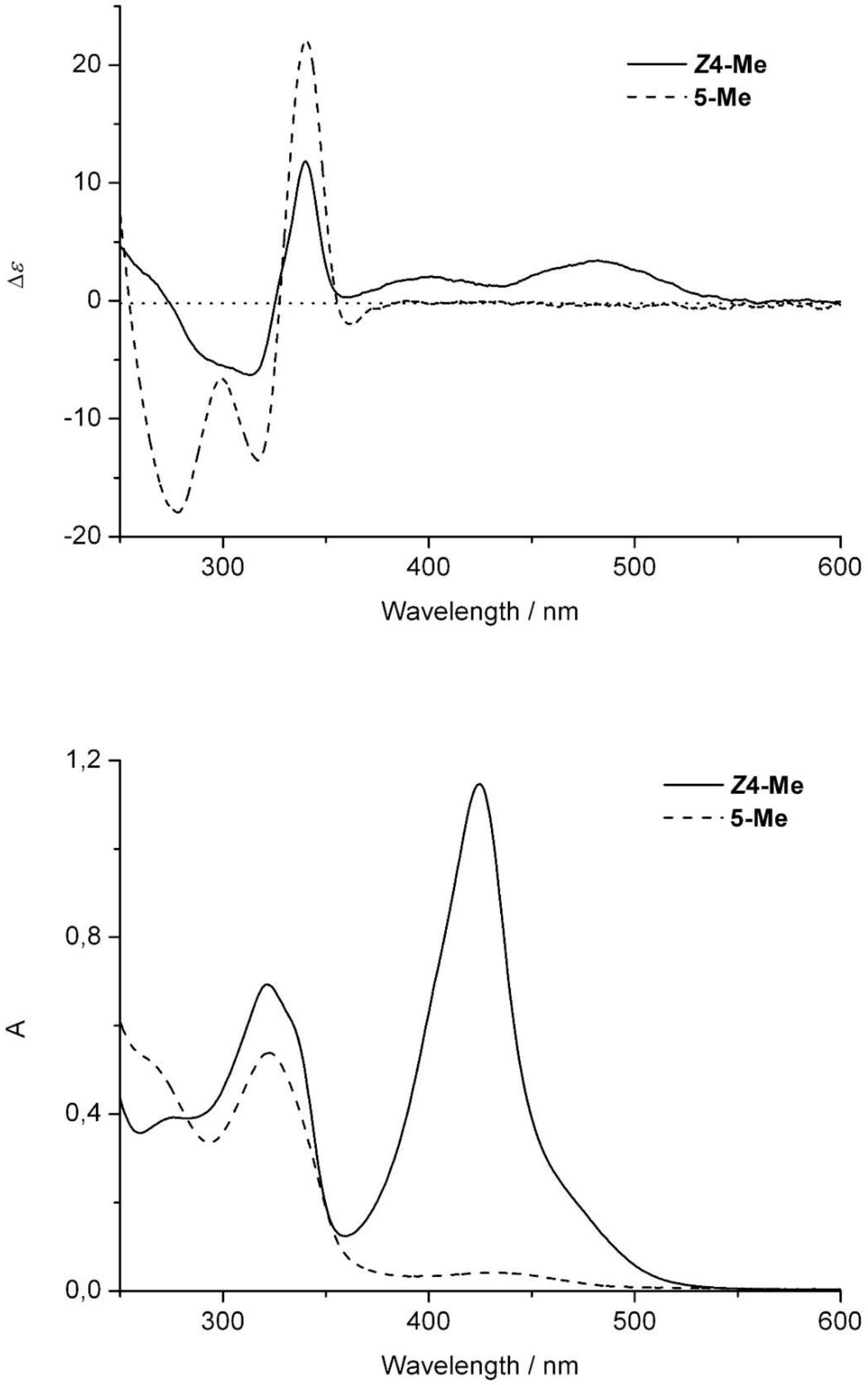

Figure S13. CD-spectra (top) of ***Z*4-Me** (2.8×10^{-4} M) and **5-Me** (1.4×10^{-4} M) and UV/Vis-spectra (bottom) of ***Z*4-Me** (2.8×10^{-4} M) and **5-Me** (1.4×10^{-4} M**)** in $CHCl_3$**.**

Thermolysis of 5-Me to *Z*4-Me

A stock solution (1.78 × 10^{-4} mol/L) of ***Z*4-Me** in acid free $CHCl_3$ was prepared. A 1 mm UV/Vis cell was filled with 0.2 mL of the stock solution and purged with Ar for 1 minute. The solution was then irradiated at 0 °C by the fluorescence lamp for 120 minutes, until the absorption at 420 nm decreased no further in the absorption spectra. Subsequently, the solution of **5-Me** was left at 23 °C in darkness. The absorption of the solution at 420 nm was determined over the time 10 h to monitor the reversion of **5-Me** to ***Z*4-Me**. Two parallel experiments were also performed to monitor the decomposition reaction of **5-Me** to ***Z*4-Me** at 40 °C, and 50 °C, respectively. The absorption at 420 nm was monitored in order to analyse the decomposition kinetics (see Figure 10 in the main part).

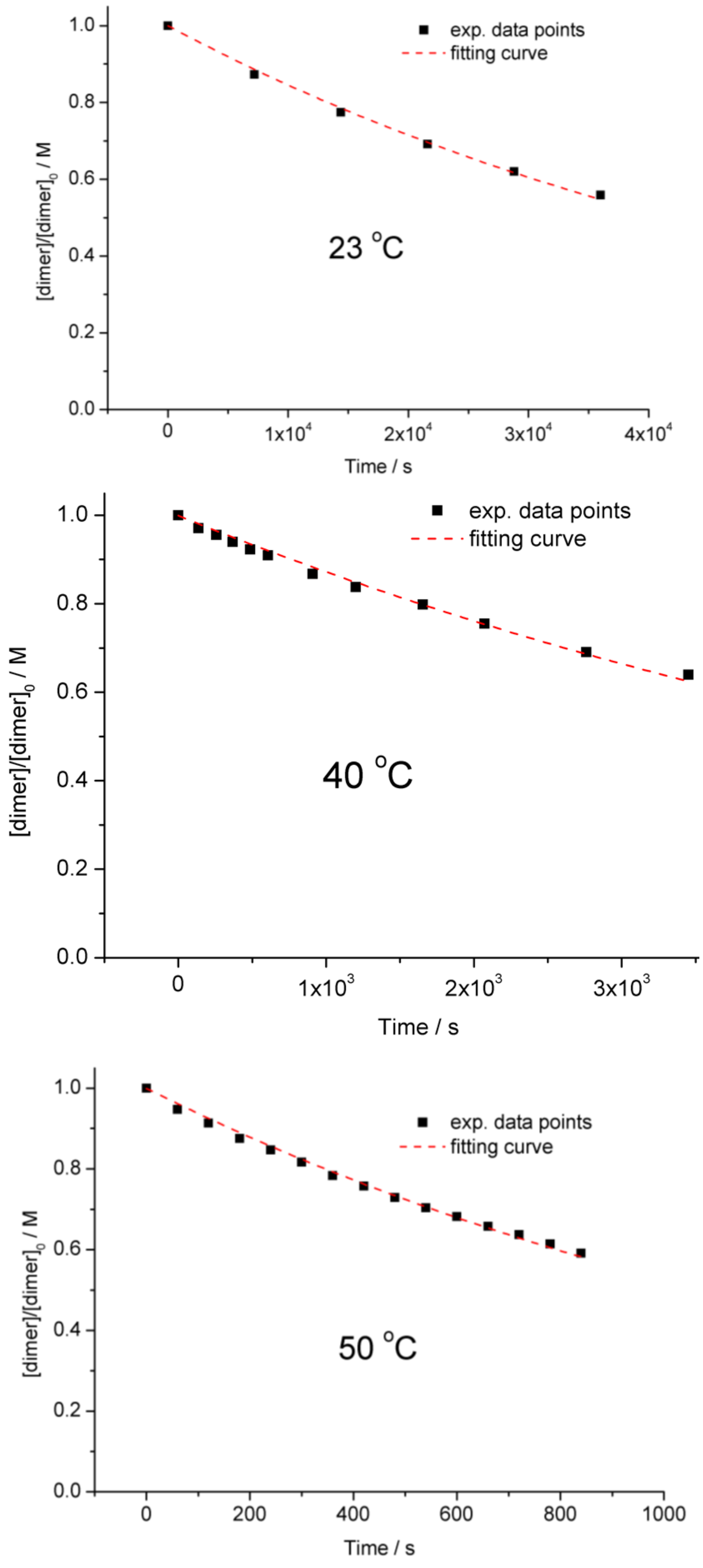

Figure S14. Kinetic analysis of the thermal decomposition of **5-Me** (8.9 × 10^{-5} M) in Ar-purged acid free $CHCl_3$ in the dark and at 23°C (top), 40°C (middle), 50°C (bottom) - k values were obtained by fitting the experimental data with the equation of $[A] = [A]_0\, e^{(-kt)}$.

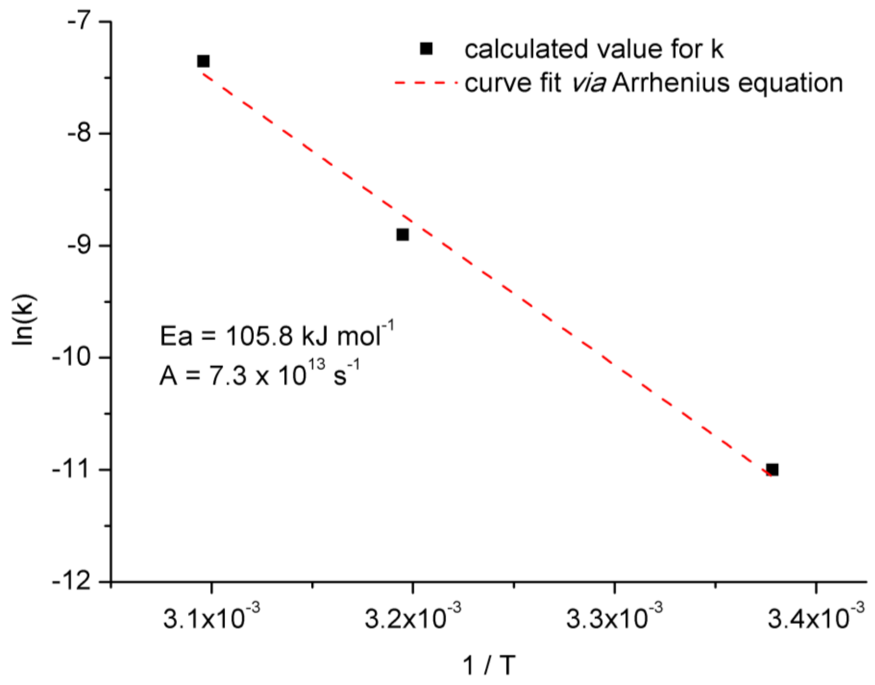

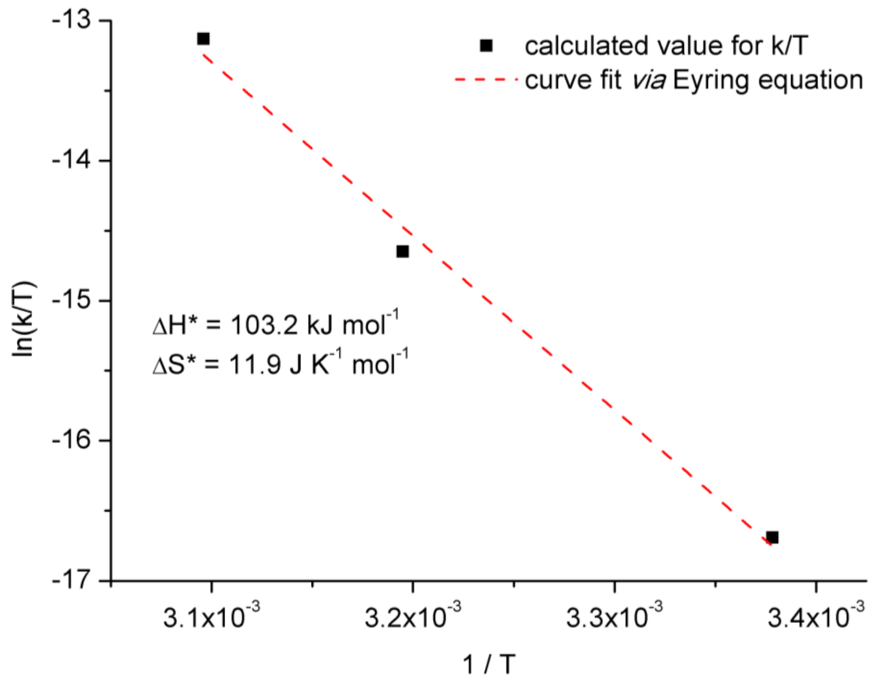

Figure S15. Determination of the activation parameters for the thermolysis of dimer **5-Me** (8.9×10^{-5} M) in Ar-purged, acid free $CHCl_3$. Top. Calculation of Ea (activation energy) and A (frequency factor) using the Arrhenius equation. Bottom. Calculation of ΔH* (the enthalpy of activation) and ΔS* (entropy of activation) using the Eyring equation on the basis of *k* values determined at different temperatures for the [2+2] cycloreversion of dimer **5-Me**.

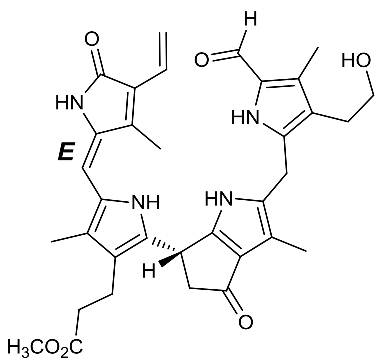

***E*4-Me**

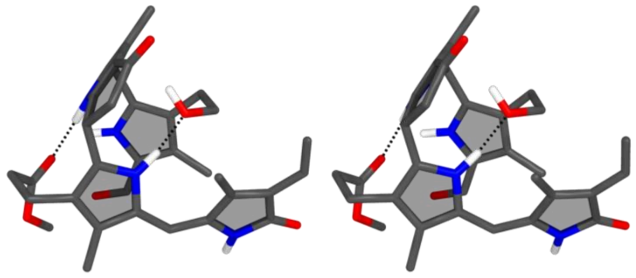

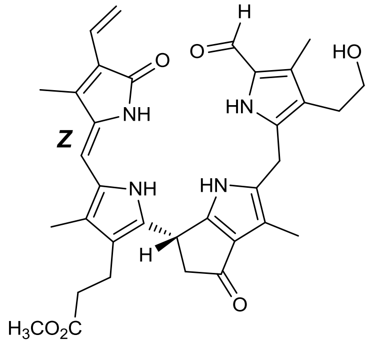

***Z*4-Me**

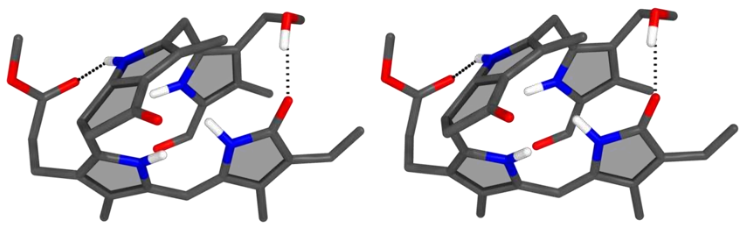

Figure S16. Stereo-projection of two calculated models of the ***E*4-Me** (top) and ***Z*4-Me** (bottom) - gas phase calculations of monomeric structures (dashed line: H-bonds; color code: C gray, O red, N blue, H white).

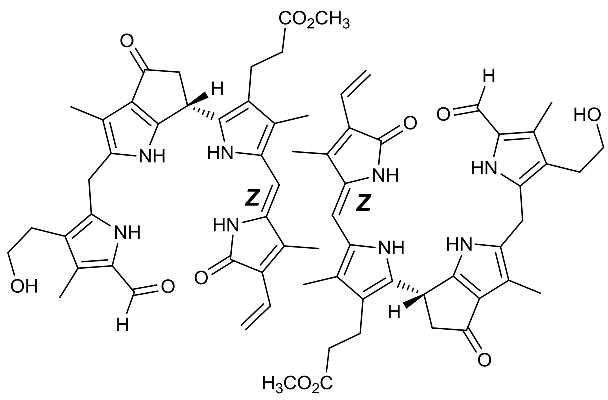

(*Z*4-Me)$_2$

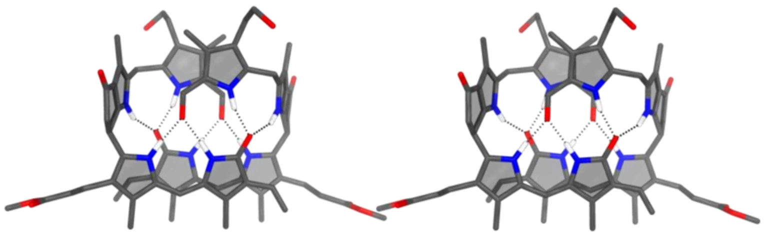

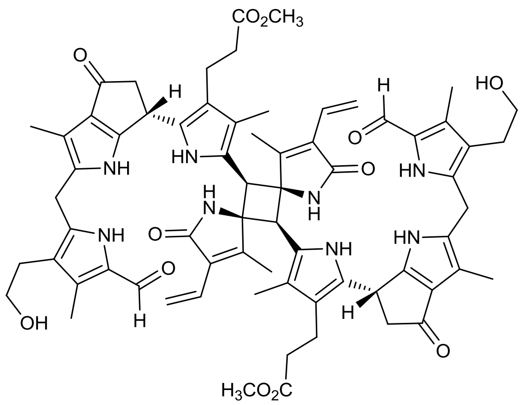

5-Me

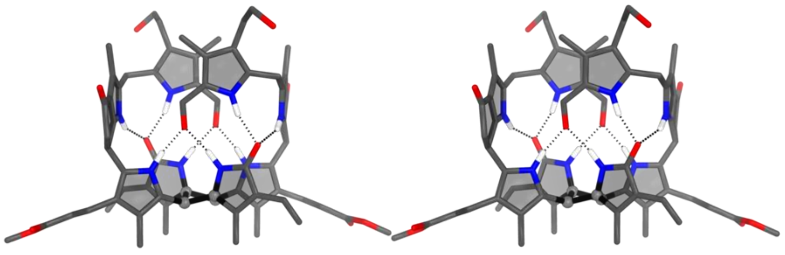

Figure S17. Stereo-projection of two calculated models of the homo-dimeric structure of **(*Z*4-Me)$_2$** (top) and of **5-Me** (bottom) (dashed line: H-bonds; color code: C gray, O red, N blue, H white).

Table S5: Structural parameters (bond lengths, π-π stacking distances C16-C16', and angles between ring planes) of the two crystallographic structures of **(*Z*4-Me)$_2$** and **(YCC-Me)$_2$**, as well as of the calculated structures of H-bonded homo dimers **(*Z*4-Me)$_2$** and **(YCC-Me)$_2$** and of the respective photo-dimers **5-Me** and **6-Me**. Bond lengths are given in Å and dihedral angles in °.

	**(Z4-Me)$_2$**	**5-Me**	**Exp.**	**(YCC-Me)$_2$**	**6-Me**	**Exp.**
C15-C16'	3.6001	1.6323	3.9757	3.5966	1.6154	3.6436
C16-C15'	3.6624	1.6305	3.9841	3.7740	1.6160	3.8215
C15-C16	1.3762	1.5587	1.3185	1.3751	1.5667	1.3505
C16'-C15'	1.3758	1.5580	1.3887	1.3773	1.5667	1.3705
C16-C16'	3.2802		3.6132	3.3188		3.3689
C1-C1'	3.310	3.367	3.400	3.280	3.297	3.474
C2-C2'	3.215	3.267	3.586	3.578	3.397	3.810
B-E	5.4	5.2	5.3	7.7	5.3	5.7
B'-E'	7.8	5.0	3.3	5.2	5.2	4.6
B-CD	64.0	62.1	57.2	58.4	56.0	67.4
B'-C'D'	64.0	61.2	57.5	62.0	55.0	62.0
E-CD	68.7	66.8	61.0	54.8	61.0	72.1
E'-C'D'	71.0	65.7	57.1	66.2	60.7	64.5
13-12-12^1-12^2	-78.1	-83.2		80.1	80.2	
13'-12'-$12^{1'}$-$12^{2'}$	-79.8	-80.7		-107.6	80.1	

Calculated with BP86/def2-TZVP/D3

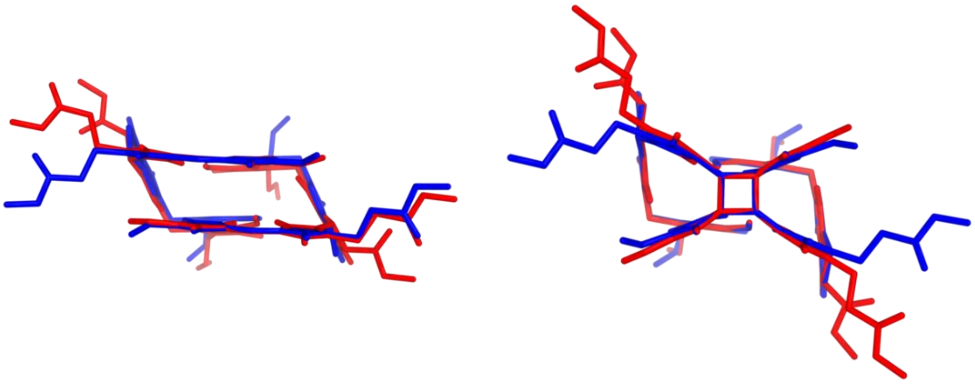

Figure S18. Overlay of the BP86/RI/def2-TZVP/D3 optimized structures. Left. Overlay of **(*Z*4-Me)$_2$**, depicted in blue, and **(YCC-Me)$_2$**, depicted in red. Right. Overlay of the photo-dimer **5-Me** (blue color) with the **YCC-Me** photo-dimer **6-Me** (red color), both structures fitted at their cyclobutane cores. Data for **(YCC-Me)$_2$** and for its photo-dimer **6-Me** are taken from Li et al. *Angew. Chem Int. Ed*. **2016**, *55*, 15760-15765.
